# Endocardial TIE2 orchestrates cardiac trabeculation via sequestering FOXO1 within the cytoplasm and stimulating versican cleavage

**DOI:** 10.1242/dev.205674

**Published:** 2026-07-29

**Authors:** H. Scott Baldwin, Kevin Tompkins, Yan Gong, Bryan Helm, Qi Liu, Xianghu Qu

**Affiliations:** ^1^ Department of Pediatrics (Cardiology), Vanderbilt University Medical Center, Nashville, TN 37232, USA; ^2^ Department of Cell and Development Biology, Vanderbilt University, Nashville, TN 37232, USA; ^3^ Department of Biostatistics, Vanderbilt University Medical Center, Nashville, TN 37232, USA

**Keywords:** Cardiac trabeculation, Endocardial cells, TIE2, FOXO1, Timp3, Versican, Mouse

## Abstract

Abnormal cardiac trabeculation often leads to congenital heart disease (CHD). TIE2-ANG1 signaling is a key regulator of endocardial growth during trabeculation. However, little is known about the underlying mechanisms. Here, we demonstrate that loss of endocardial *Tie2* via *Nfatc1^Cre^* resulted in increased nuclear localization of forkhead box O1 (FOXO1), reduced phosphorylation of AKT (the direct regulator of FOXO1) and reduced expression of several proliferation promoting genes in endocardial cells (EdCs). The cardiac defects seen in endocardial *Tie2-*deficient mouse hearts were phenocopied by forced activation of FOXO1 within the endocardium and partially rescued by loss of *Foxo1*. Conversely, excessive PI3K signaling (the direct regulator of phosphorylation of AKT) in EdCs caused cardiac hypotrabeculation. Further, we identify that endocardial loss of *Tie2* resulted in impaired cleavage of versican, which was associated with enhanced expression of ADAMTS endogenous inhibitor TIMP3 in *Tie2-cko* EdCs. Thus, our results suggest that TIE2 signaling in the early embryonic endocardium promotes endocardial growth via sequestering FOXO1 within the cytoplasm and modulates cardiomyocyte proliferation via stimulating versican cleavage to orchestrate myocardial trabeculation, providing insights into the pathogenesis of CHD.

## Teaser

**Highlighted Article:** Endocardial TIE2 signaling in embryonic heart promotes endocardial growth via sequestering FOXO1 within the cytoplasm and modulates myocardial trabeculation via stimulating versican cleavage through FOXO1-dependent inhibition of ADAMTS1 by TIMP3.

## INTRODUCTION

Congenital heart diseases (CHDs) are the most common birth defects and are primarily caused by genetic mutations that lead to impaired embryonic heart development *in utero* (Nees and Chung, 2020; Samsa et al., 2013). During heart development, reciprocal molecular communication between the endocardial cell (EdC) and cardiomyocyte (CM) layers is essential for myocardial trabeculation, valve and ventricular chamber formation. Endocardial cues orchestrate CM proliferation, survival and organization. Additionally, the endocardium enables oxygenated blood to reach the CMs, which, in turn, secrete factors that promote endocardial growth and function. Misregulation of this delicate and complex endocardial-myocardial interplay can result in CHDs, such as left ventricular noncompaction cardiomyopathy, congenital valve diseases and heart failure (Choquet et al., 2019; Dye and Lincoln, 2020; Liu et al., 2018b; Rojanasopondist et al., 2022). These processes are poorly understood, therefore further delineation of underlying genetic and molecular factors will be vital in the development of therapies to promote cardiac homeostasis and regeneration.

EdCs are a highly specialized subset of endothelial cells (ECs), distinct from vascular ECs, that form the innermost layer of the heart wall. Specification of EdCs and other ECs from mesodermal precursors is induced by bone morphogenetic proteins and WNT inhibitors (Marvin et al., 2001; Schultheiss et al., 1997) and is regulated by VEGFR2 (also known as KDR; Sakurai et al., 2005; Schultheiss et al., 1997; Shalaby et al., 1995) and ETV2 (Gong et al., 2022; Ferdous et al., 2009; Koyano-Nakagawa et al., 2012) signaling pathways. However, little is known about how endocardial growth is regulated after EdC specification, especially during cardiac trabeculation.

We have identified TIE2 (tyrosine kinase with immunoglobulin-like and EGF-like domains 2; TEK) (Augustin et al., 2009; Eklund et al., 2017) as a key regulator of EdC proliferation during the critical window for trabeculation in mice, because loss of EdC *Tie2* via *Nfatc1^Cre^* results in embryonic heart failure characterized by impaired endocardial growth, hyperplastic trabeculation and noncompaction of ventricular myocardium (Qu et al., 2019). In addition, TIE2 is implicated in ventricular septal defect formation (Szot et al., 2018; Wouters et al., 2010). However, the mechanisms of TIE2-mediated CHDs or TIE2 signaling during trabeculation remain poorly understood. Here, we present new data showing that endocardial deletion of *Tie2* is associated with increased nuclear localization of forkhead box O1 (FOXO1, a transcription factor essential for regulation of endothelial growth in vasculature) (Menghini et al., 2020; Wilhelm et al., 2016), reduced phosphorylation of AKT (pAKT) and reduced expression of several proliferation promoting proteins in EdCs, including cyclin D1, cMYC (Myc) and phosphorylation of Yes-associated protein (YAP). The cardiac defects seen in EdC *Tie2-*deficient hearts were phenocopied by a FOXO1 gain-of-function model, *FOXO1^CA^*, wherein FOXO1 is constitutively active within the endocardium and partially rescued by loss of *Foxo1*. Furthermore, excessive PI3K signaling in EdCs resulted in the opposite effect of reduced cardiac trabeculation. Moreover, our single-cell RNA sequencing (scRNAseq) on control and *Tie2* conditional knockout (*Tie2-cko*) embryonic hearts and further immunohistochemistry and western blot analyses revealed that endocardial loss of *Tie2* resulted in impaired cleavage of versican (Vcan), an extracellular matrix (ECM) protein promoting CM proliferation (Cooley et al., 2012; Mjaatvedt et al., 1998; Nandadasa et al., 2014; Yamamura et al., 1997), which was associated with enhanced expression of the ADAMTS endogenous inhibitor TIMP3 in *Tie2-cko* EdCs. Our chromatin immunoprecipitation (ChIP) assay further confirmed that FOXO1 can bind an enhancer region located between exon 1 and exon 2 of the *Timp3* gene, suggesting that *Timp3* is a direct target of FOXO1 in the developing endocardium. Collectively, endocardial TIE2 signaling in the early embryonic heart promotes endocardial growth via sequestering FOXO1 within the cytoplasm and modulates CM proliferation via stimulating Vcan cleavage to orchestrate myocardial trabeculation, highlighting the central role of the TIE2-PI3K/AKT-FOXO1 signaling axis in heart development.

## RESULTS

### FOXO1 is a target of TIE2 activation in the endocardium

**Fig. 1.**
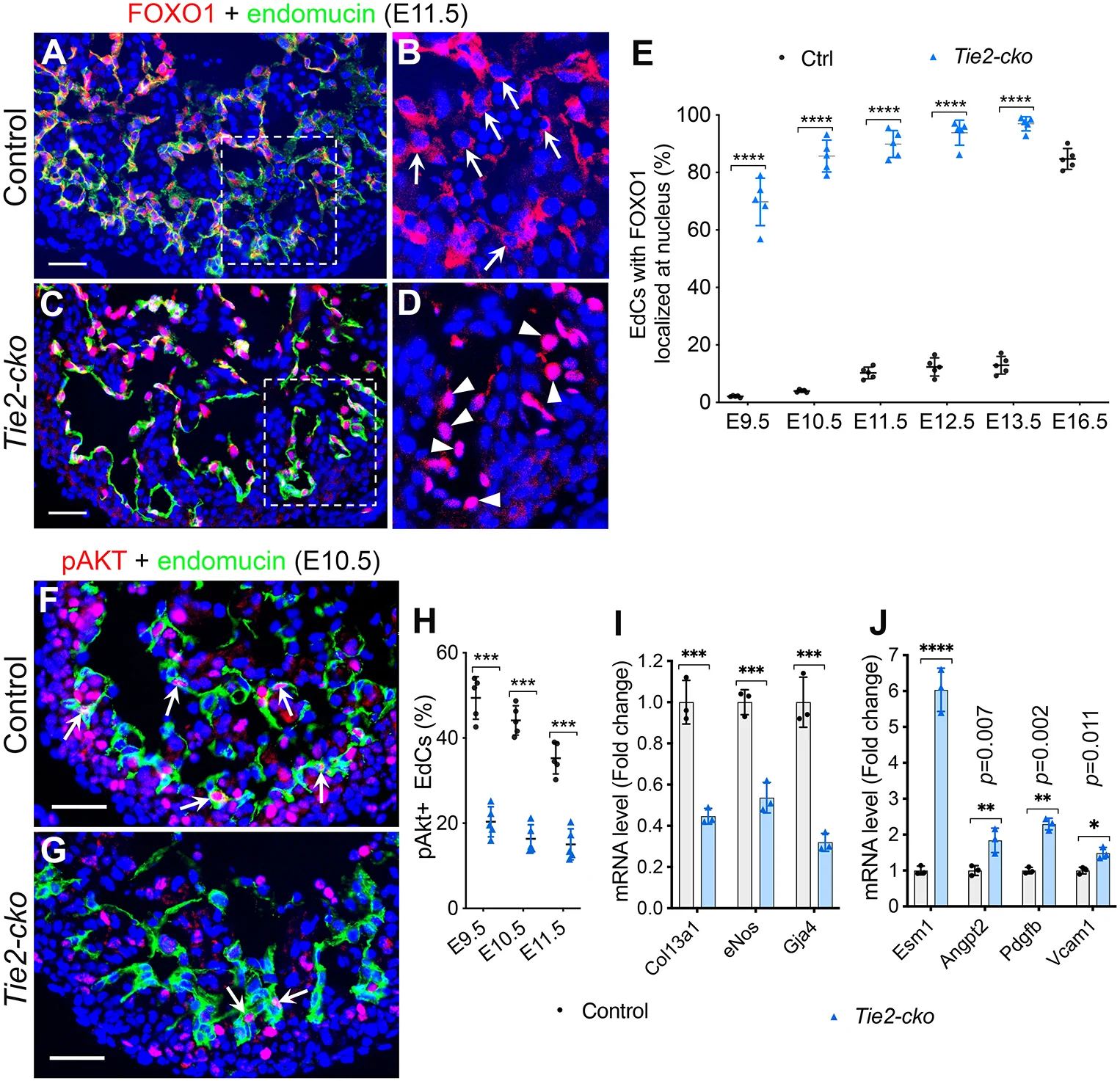
**Endocardial loss of *Tie2* resulted in nuclear import of FOXO1, allowing for FOXO1-mediated transcription regulation.** (A-D) Control (*Tie2^+/f^* and *Tie2^f/f^*) and *Tie2-cko* left ventricular sections at E11.5 were stained with FOXO1 (red) and endomucin (endocardial marker, green) antibodies. (B,D) are the higher magnification images (omitting the green channel) of the boxed areas in A and C showing a diffuse nucleocytoplasmic localization of FOXO1 in control EdCs (arrows, B), but accentuated nuclear localization in the mutant (arrowheads, D). (E) Quantification of EdCs with FOXO1 localized at nucleus (%). Note the mild gradual increase in nuclear import of FOXO1 of wild-type EdCs at E9.5-E13.5 and, by E16.5, the majority of wild-type EdCs demonstrate FOXO1 localized at nucleus. (F,G) Control and *Tie2-cko* ventricular sections at E10.5 were stained with pAKT (red) and endomucin (green) antibodies, showing reduced phosphorylation of AKT (arrows) in *Tie2-cko* EdCs compared to the control. Note more intense endomucin staining in *Tie2-cko* EdCs than in control, although their endocardial network was simplified. DAPI was used to counterstain nuclei in all images. (H) Quantification of pAKT^+^ EdCs (%). (I,J) qPCR analysis of E11.5 hearts. *n*=3 per group. Data represent the mean±s.e.m. Two-way ANOVA in E,H and two-tailed unpaired Student's *t*-test in I,J; *P<0.05; **P<0.01; ***P<0.001; ****P<0.0001. Scale bars: 50 μm.

Loss of EdC *Tie2* via *Nfatc1^Cre^* led to impaired endocardial growth due to reduced proliferation of EdCs (Qu et al., 2019), but the underlying mechanism is unknown. FOXO1 transcription factor, an enriched FOXO family member in the endothelium, is reported as a gatekeeper of endothelial growth. It is an effector of the PI3K/AKT pathway that links growth and metabolism (Brent et al., 2008; Huang and Tindall, 2007; Menghini et al., 2020; Wilhelm et al., 2016). ANG1-TIE2 has been shown to be a mitogenic signal that is mediated by AKT phosphorylation of FOXO1. resulting in nuclear exclusion in vascular ECs both *in vitro* and *in vivo* (Daly et al., 2004; Korhonen et al., 2016). Using immunofluorescence analysis, we demonstrate that FOXO1 is always co-localized with TIE2 in the mouse embryonic heart. It is highly expressed in the endocardium as early as TIE2, and it similarly has much higher expression in the endocardium than in coronary vessels (Fig. 1A; Fig. S1A). Immunohistochemistry analysis of embryonic day (E) 11.5 hearts of control and *Tie2* conditional knockout *Nfatc1^Cre^;Tie2^f/f^* with a knock-in *Nfatc1^Cre^* (referred to as *Tie2-cko*) revealed a diffuse nucleocytoplasmic localization of FOXO1 in control EdCs, but predominant nuclear localization in the mutants (Fig. 1A-D). Further immunohistochemistry analyses and quantification revealed that the increase in nuclear import of FOXO1 in *Tie2-cko* EdCs persisted during the entire period of trabeculation (E9.5-E13.5) (Fig. 1E; Fig. S1B). Consistently, pAKT, the regulator upstream of FOXO1 but downstream of TIE2 signaling in vascular ECs (Augustin et al., 2009; Eklund et al., 2017), was dramatically reduced over time in *Tie2-cko* EdCs (Fig. 1F-H; Fig. S2A).

**Fig. 2.**
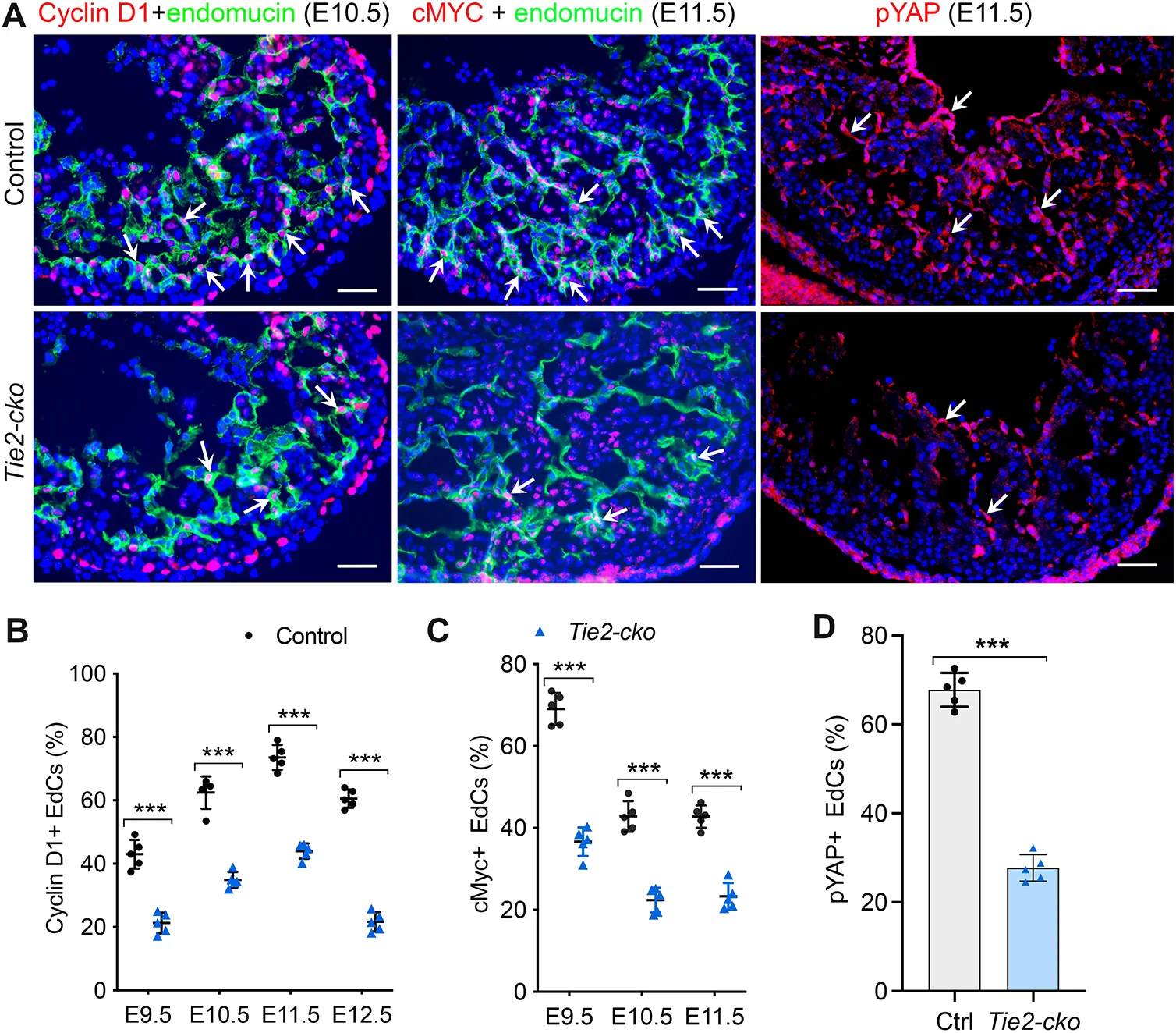
**Endocardial loss of *Tie2* led to reduced expression of several proliferation promoting genes in EdCs.** (A) Control and *Tie2-cko* left ventricular sections were stained with cyclin D1, cMYC, pYAP and endomucin antibodies. Arrows indicate representative positive staining in EdCs. DAPI was used to counterstain nuclei in all images. (B-D) Quantification of cyclin D1^+^ EdCs (B), cMYC^+^ EdCs (C) and pYAP^+^ EdCs (D). Data represent the mean±s.e.m. Two-way ANOVA in B,C and two-tailed unpaired Student's *t*-test in D; ***P<0.001. Scale bars: 50 μm.

In agreement with the above findings, our transcriptome analysis from the bulk RNAseq of control and *Tie2-cko* E11.5 hearts, and further confirmation by quantitative RT-PCR (qPCR), revealed that a group of target genes [*Col13a1*, *eNos* (*Nos3*) and *Gja4*], normally downregulated by nuclear FOXO1 (Paik et al., 2007; Potente et al., 2005), had decreased expression in *Tie2-cko* hearts (Fig. 1I) consistent with increased nuclear access of FOXO1 in the absence of TIE2 inhibition. Similarly, a cadre of genes normally upregulated by FOXO1 (*Esm1*, *Ang2*, *Pdgfb* and *Vcam1*) (Paik et al., 2007; Papanicolaou et al., 2008; Potente et al., 2005) demonstrated increased expression in mutant hearts (Fig. 1J) consistent with accentuated nuclear access of FOXO1 resulting from attenuated TIE2 signaling. Furthermore, immunohistochemistry analyses revealed that expression of cyclin D1 and cMYC was reduced in *Tie2-cko* EdCs compared with control, although no significant changes were detected in CMs (Fig. 2A,B; Fig. S2B). Cyclin D1 is a key regulator of cell proliferation that promotes the transition from G1 to S phase of the cell cycle, and it is suppressed by FOXO1 (Huang and Tindall, 2007; Riddell et al., 2018). cMYC has been shown to be an essential effector of FOXO1 in coordination of angiogenic endothelial metabolism and growth (Riddell et al., 2018; Wilhelm et al., 2016). Quantification of cMYC expression in EdCs revealed that cMYC abundance is gradually reduced in the endocardium of control hearts during the period of trabeculation (Fig. 2C), coinciding with decreasing EdC proliferation rate evaluated via co-staining of BrdU-ERG or Ki67-ERG (Fig. S3A-D) and the gradual increase in nuclear import of FOXO1 (Fig. 1E). This suggests that cMYC might be a crucial driver of EdC proliferation and growth in the developing endocardium during trabeculation. In addition, endocardial loss of *Tie2* led to reduced phosphorylation of YAP in *Tie2-cko* EdCs, compared with the control (Fig. 2A,D), although no significant changes were detected on YAP expression (Fig. S4). Hippo-YAP/TAZ signaling plays pivotal roles in regulating cell proliferation, growth and survival (Xiao et al., 2018; Xin et al., 2011). Thus, the above results suggest that endocardial TIE2 signaling in early embryonic heart promotes endocardial growth via sequestering FOXO1 within the cytoplasm.

### Cardiac defects in *Tie2-cko* mutants were phenocopied by endocardial forced activation of FOXO1 and partially rescued by loss of *Foxo1*

**Fig. 3.**
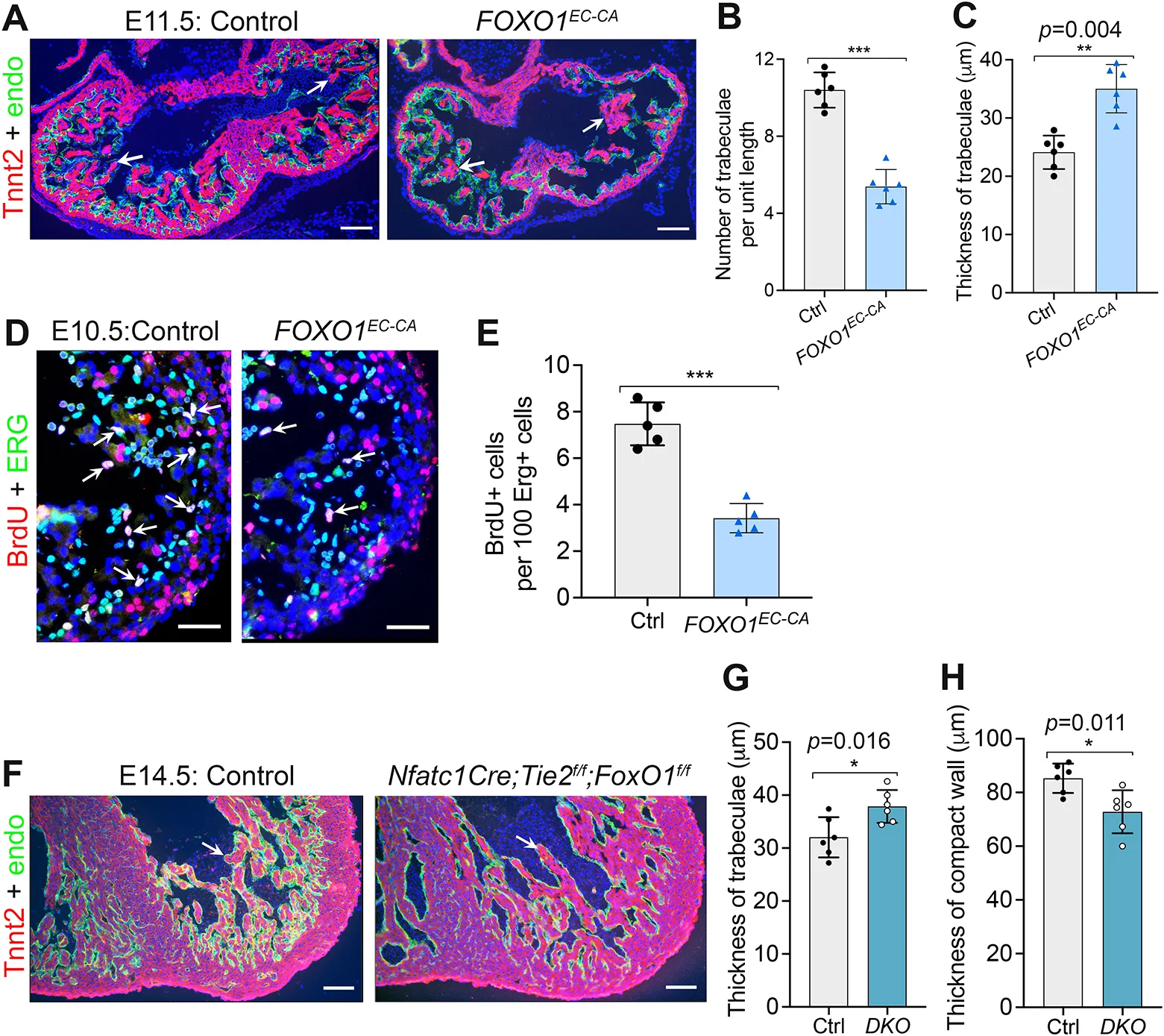
**Cardiac defects in *Tie2-cko* mice were phenocopied by endocardial forced activation of FOXO1 and partially rescued by loss of *Foxo1*.** (A) Control (*FOXO1^CA/+^*) and *FOXO1^EC-CA^* ventricular sections at E11.5 were stained for troponin T (Tnnt2, myocardial marker) and endomucin (endo), showing abnormal trabeculae (arrows) and endocardial network in the mutant heart. (B,C) Quantification of trabeculae density and trabeculae thickness at E11.5. (D) BrdU pulse labeling and co-immunostaining of ventricular sections of control and *FOXO1^EC-CA^* embryos at E10.5 with BrdU (red) and ERG (green, EC marker). The cells positive for both BrdU and ERG represent proliferating EdCs (arrows). (E) Quantification of BrdU^+^/ERG^+^ cells as a percentage of total ERG^+^ cells in endocardium. (F) Control (*Tie2^f/+^;Foxo1^f/f^* and *Tie2^f/f^;Foxo1^f/f^*) and *Nfatc1^Cre^;Tie2^f/f^;Foxo1^f/f^* heart sections at E14.5 were stained with antibodies for troponin T and endomucin. Arrows indicate trabeculae. (G,H) Quantification of trabeculae thickness (G) and thickness of compact wall (H) at E14.5. DAPI was used to counterstain nuclei in all images. Data represent the mean±s.e.m. Two-tailed unpaired Student's *t*-test; *P<0.05; **P<0.01; ***P<0.001. Scale bars: 50 μm (D); 100 μm (A,F).

To further determine if TIE2 signaling modulates EdC proliferation via regulation of FOXO1 in the developing endocardium, we used a Cre-inducible, gain-of-function allele *FOXO1^CA^* in which the three AKT phosphorylation sites are mutated, thus preventing AKT phosphorylation and ‘locking’ FOXO1 in the nucleus (Huang and Tindall, 2007; Menghini et al., 2020). This *FOXO1^CA^* allele was generated by inserting a cDNA fragment coding for a haemagglutinin (HA)-tagged human *FOXO1* mutant [HA-*hFOXO1* (AAA)], preceded by a loxP-flanked ‘STOP’ cassette into the *Rosa26* locus (Ouyang et al., 2012). This forced activation of *FOXO1* allele was bred onto an EdC-specific *Nfatc1^Cre^* line to generate the internal ribosomal entry site (IRES)-GFP-coexpressing mutants. In the resultant experimental model (*Nfatc1^Cre^;FOXO1^CA/+^*, hereby referred to as *FOXO1^EC-CA^*), 31.6% (12/38) of mutant embryos developed pericardial effusions and died before E13.5, while their survival rates at E14.5-E15.5 and at E16.5-E18.5 were 42.5% and 30.4%, respectively (Table S3); only two *FOXO1^EC-CA^* mutants from over nine litters (*FOXO1^CA/CA^*×*Nfatc1^Cre^*) survived to birth. Dual-fluorescence staining of mutant heart sections revealed a simplified endocardial network with reduced EdC number, abnormal trabeculation (fewer but thicker trabeculae) and a thinner compact myocardium in mutants, compared with littermate controls (Fig. 3A-C; Fig. S5A-D). Accordingly, BrdU incorporation assay revealed that the EdC proliferation rate in *FOXO1^EC-CA^* hearts at E10.5 was reduced by 53.3% (Fig. 3D,E), but trabecular CM proliferation rate in *FOXO1^EC-CA^* hearts at E11.5 was increased by 24.6% (Fig. S5E-G), when compared to controls, as in *Tie2-cko* (Qu et al., 2019). Immunostaining of mutant heart sections for FOXO1 confirmed accentuated nuclear localization of FOXO1 in EdCs of *FOXO1^EC-CA^* hearts, indicating successful overexpression of FOXO1 (Fig. S5H). Thus, the cardiac defects seen in *Tie2-cko* mice were phenocopied in these *FOXO1^EC-CA^* mutants. In our attempts to determine why about half of *FOXO1^EC-CA^* mutants died before E13.0 while another half could survive beyond E13.5, we performed immunohistochemistry of *FOXO1^EC-CA^* heart sections at E10.5 with GFP (as a surrogate for FOXO1 overexpression). This revealed variations in the efficiency of FOXO1 overexpression in ventricular EdCs of different embryos: there were more than 50% of GFP^+^ EdCs in some mutants but as low as only about 10% in others (Fig. S5I,J).

To further directly test if endocardial *Tie2*-mediated exclusion of FOXO1 from the EdC nucleus is a primary driver of the endocardial growth phenotype during trabeculation, we combined *Foxo1^fl/fl^*, *Tie2^fl/fl^* and *Nfatc1^Cre^* alleles to generate EdC-specific double mutants (*Nfatc1^Cre^;Tie2^f/f^;Foxo1^f/f^*), which should result in a rescued phenotype. First, we crossed a floxed *Foxo1* allele (Paik et al., 2007) with an *Nfatc1^Cre^* line to delete *Foxo1* in endocardium from E9.0, the resulting conditional knockout mice (*Nfatc1^Cre^;Foxo1^f/f^*) had a normal lifespan with normal endocardial growth and trabeculation (Fig. S6A-D), although mild lymphatic defects (such as increased lymphatic valve number) were observed, as previously reported (Baldwin et al., 2025). Immunostaining revealed that *Nfatc1^Cre^*-mediated FOXO1 deletion was efficient in *Nfatc1^Cre^;Foxo1^f/f^* endocardium (Fig. S6E,F). Female *Tie2^f/f^;Foxo1^f/f^* were then crossed with male *Nfatc1^Cre^;Tie2^f/+^;Foxo1^f/f^* to make double knockout *Nfatc1Cre;Tie2^f/f^;Foxo1^f/f^* animals. Surprisingly, the majority of double knockout embryos died at E13.5 due to abnormal trabeculation similar to *Tie2-cko* embryos, which never survived beyond E13.5 (Qu et al., 2019). Only 25% (5 out of 20) of the double knockout embryos survived to E14.5-E15.5, and none survived to birth. Histological analysis and immunostaining of heart sections revealed that the surviving double knockout embryos also displayed a simplified endocardial network (visualized by staining with endomucin) and thicker trabeculae, compared with littermate controls (Fig. 3F-H). However, their phenotypes were not as severe as in *Tie2-cko* hearts (Qu et al., 2019). Thus, these results suggest a partial rescue of the trabecular phenotype resulting from attenuation of TIE2-mediated nuclear exclusion of FOXO1.

Because endocardial deletion of *Tie2* resulted in FOXO1 nuclear localization, which was associated with a significant reduction of cMYC expression in EdCs, the endocardial phenotypes imposed by FOXO1 activation could be rescued by restoring cMYC signaling. To test this directly, we used a Cre-mediated *cMYC* overexpressor allele (*cMYC^OE^*), which was generated by inserting human *cMYC* cDNA, preceded by a loxP-flanked STOP cassette and marked by a signaling-deficient truncated version of human *CD2* under the control of an IRES downstream of the inserted cDNA, into the *Rosa26* locus. Transgene transcription is controlled by the CAG promoter (Sander et al., 2012). First, we crossed a floxed c*MYC^OE^* allele with a *Nfatc1^Cre^* line to generate an endocardial-specific cMYC overexpression model. The resulting mutant mice (*Nfatc1^Cre^;MYC^OE/+^*) displayed normal endocardial growth and trabeculation, although they developed edema later due to defective lymphatic development as previously described (Sander et al., 2012). Dual-fluorescence staining of heart sections with antibodies for cMYC and endomucin confirmed successful and efficient overexpression of cMYC specifically in the endocardium (Fig. S7A,B). Then, we combined the *cMyc^OE^*, *Tie2^f/f^* and *Nfatc1^Cre^* alleles to generate endocardial-specific double mutant *Nfatc1^Cre^;Tie2^f/f^;Myc^OE/+^*. All of the resultant double mutant embryos died at the same time as *Tie2-cko* and displayed the same cardiac defects including impaired endocardial growth and hyperplastic trabeculation (Fig. S7C,D). These results suggest that cMYC is not a major effector of endocardial TIE2-FOXO1 signaling during trabeculation.

### Endocardial FOXO1 nuclear localization *in vivo* is not dependent on VEGF-VEGFR2 signaling

**Fig. 4.**
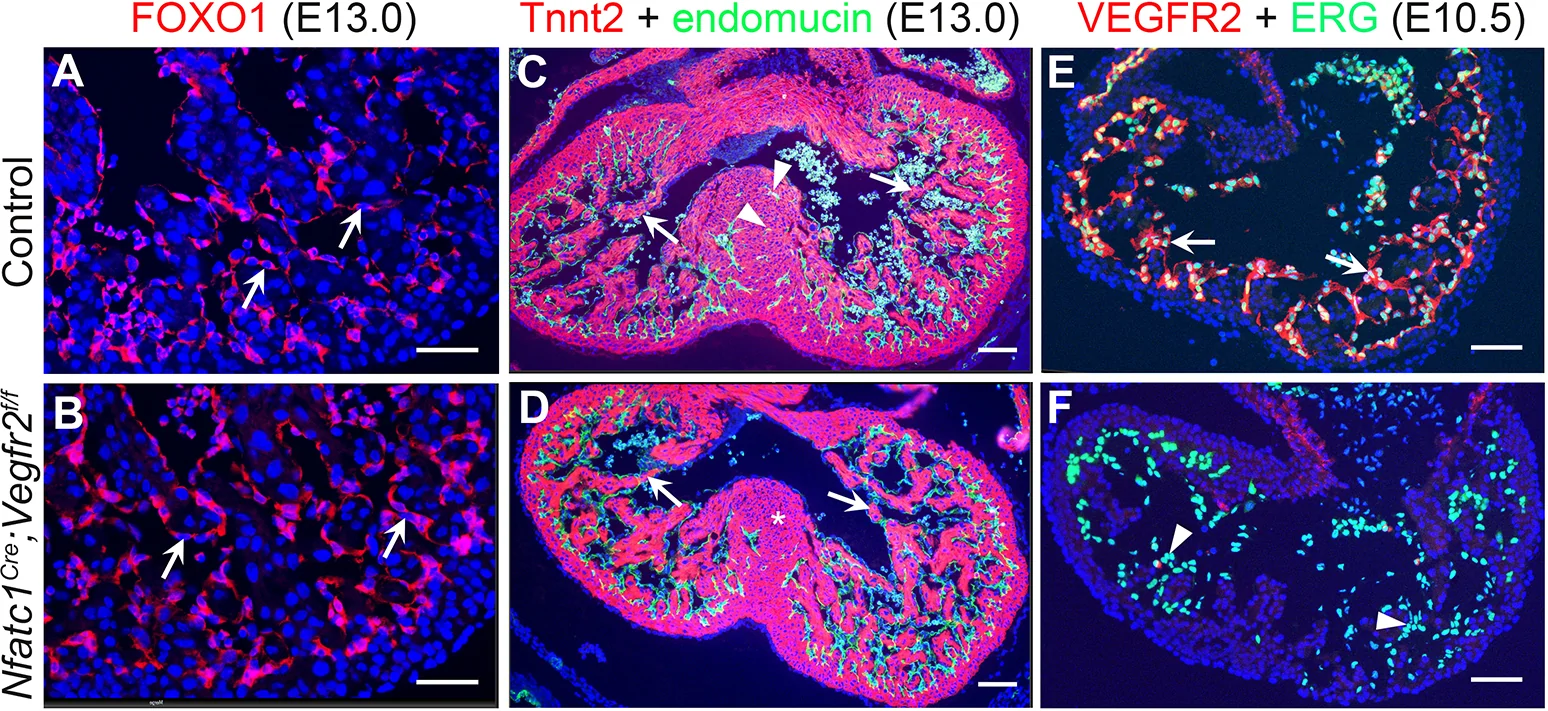
**Endocardial loss of *Vegfr2* led to defective coronary vasculature but normal trabeculation and EdC network, with normal FOXO1 localization.** (A-D) Control and *Nfatc1^Cre^;Vegfr2^f/f^* ventricular sections at E13.0 were stained with FOXO1 antibody or co-stained for troponin T (Tnnt2) and endomucin, showing normal nucleocytoplasmic localization of FOXO1 (arrows in A,B), normal trabeculae (arrows in C,D) and endocardial network in both control and mutant hearts, but severely diminished or no coronary vessels present in the ventricular septum (asterisk) of the mutant, compared to normal coronary vessels in the control (arrowheads). (E,F) Control and *Nfatc1^Cre^;Vegfr2^f/f^* ventricular sections at E10.5 were stained for VEGFR2 and ERG, showing only minimal and sporadic VEGFR2 staining (arrowheads) in the mutant endocardium, in contrast to robust staining in the control (arrows). DAPI was used to counterstain nuclei in all images. Scale bars: 50 μm (A,B); 100 μm (C-F).

Since VEGF/PI3K/AKT signaling has also been reported to promote FOXO1 cytoplasmic localization in a context-dependent manner, resulting in its inactivation in ECs *in vitro* (Abid et al., 2004; Kim et al., 2019), the VEGF-VEGFR2 pathway may also be important for endocardial growth and expansion during cardiac trabeculation. However, endocardial-specific deletion of *Vegfr2* via *Nfatc1^Cre^* did not lead to FOXO1 nuclear localization in the mutant (*Nfatc1^Cre^;Vegfr2^f/f^*) endocardium (Fig. 4A,B). In line with this, mutant hearts did not exhibit defects in trabeculation or endocardial growth, although coronary vasculature defects were detected from E12.5 as previously reported (Wu et al., 2012), with severely diminished or no coronary vessels present in the ventricular septum (Fig. 4C,D). Efficient *Nfatc1*^Cre^-mediated deletion of *Vegfr2* in the endocardium at E10.5 was confirmed by dual immunohistochemistry with VEGFR2 and ERG antibodies, which also demonstrated no change in the total number of ERG^+^ EdCs between control and mutant embryos (Fig. 4E,F), indicating normal proliferation of EdCs. These results are consistent with previous reports using a zebrafish model showing that endocardial growth during cardiac ballooning stages is not regulated by VEGF signaling (Dietrich et al., 2014; Haack and Abdelilah-Seyfried, 2016). Thus, endocardial growth/EdC proliferation during trabeculation is independent of VEGFR2 signaling.

### Excessive PI3K signaling in EdCs causes cardiac hypotrabeculation

**Fig. 5.**
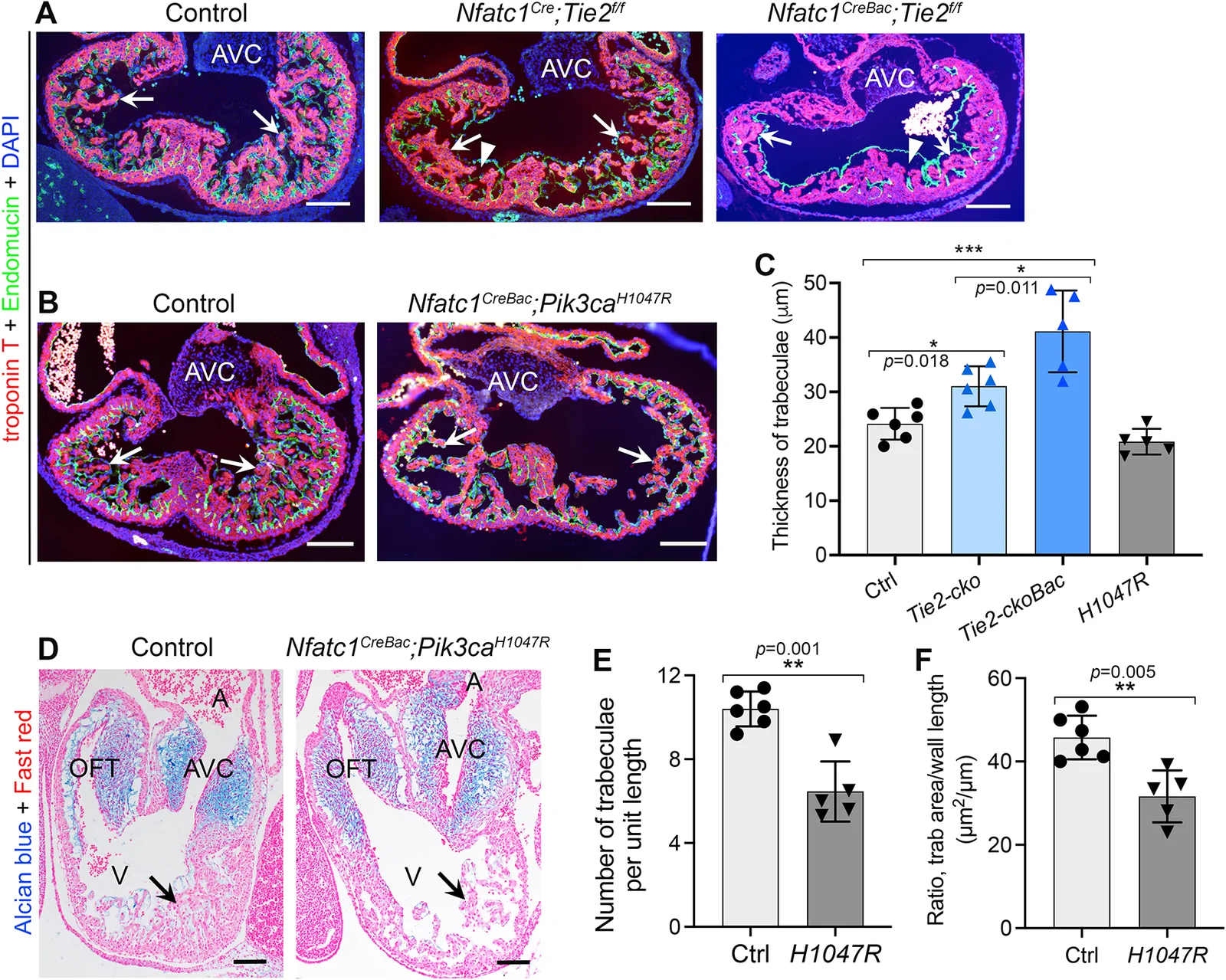
**Excessive PI3K signaling in EdCs resulted in reduced cardiac trabeculation.** (A,B) Heart sections at E11.5 were stained for troponin T and endomucin. (A) Endocardial deletion of *Tie2* via the knock-in *Nfatc1^Cre^* or the new Bac *Nfatc1^Cre^* both led to impaired endocardial network and hyperplastic trabeculae, but the cardiac defects with *Nfatc1^CreBac^* were more severe than those with the knock-in *Nfatc1^Cre^*. Note increased space between endocardium and myocardium (arrowheads) in both mutant embryos, indicating increased extracellular matrix (ECM) accumulation. (B) Endocardial overexpression of *Pik3ca^H1047R^* via *Nfatc1^CreBac^* led to reduced density of trabeculae in *Nfatc1^CreBac^;Pik3ca^H1047R^* ventricles, compared to control littermates. (C) Quantification showing the thickness of the trabecular sheet at E11.5: *Tie2-ckoBac* (*Nfatc1^CreBac^;Tie2^f/f^*)>*Tie2-cko* (*Nfatc1^Cre^;Tie2^f/f^*)>Ctrl (control) and H1047R (*Nfatc1^CreBac^;Pik3ca^H1047R^*). (D) Alcian Blue and Nuclear Fast Red staining of sagittal paraffin sections of hearts at E11.5, showing normal size of endocardial cushions but reduced trabeculae density in *Nfatc1^CreBac^;Pik3ca^H1047R^* hearts, compared to control littermates. (E,F) Quantification of the number of trabeculae and ratio of overall trabecular area and length of the compact wall based on B. Arrows indicate trabeculae. A, atrium; AVC, atrioventricular canal; OFT, outflow tract; V, ventricle. Data represent the mean ± s.e.m. One-way ANOVA in C and two-tailed unpaired Student's *t*-test in E,F; *P<0.05; **P<0.01; ***P<0.001. Scale bars: 100 μm.

The PI3K pathway, a central regulator of cell survival, growth and metabolism, plays a crucial role in vascular homeostasis, and is essential for angiogenesis and maintenance of the mature endothelium (Graupera and Potente, 2013). TIE2 signaling is upstream of PI3K signaling during cerebral cavernous malformations (Li et al., 2026) and Pik3ca^H1047R^-driven venous malformations (Kraft et al., 2025), which were rescued by genetic or pharmacologic blockade of TIE2 in mouse models. To test if PI3K signaling (downstream of TIE2 but upstream of FOXO1 in vascular ECs) is directly involved in cardiac trabeculation, we took advantage of the previously reported gain-of-function allele *R26-Pik3ca^H1047R^*. This strain carries a floxed stop cassette upstream of the H1047R mutant allele of *Pik3ca* (phosphatidylinositol 3-kinase, catalytic, alpha polypeptide; also called PI3K) within the *Gt(ROSA)26Sor* locus (Adams et al., 2011). Initially, we crossed female homozygous floxed *Pik3ca^H1047R^* mice with the knock-in *Nfatc1^Cre^* male mice. Unexpectedly, 15.8% (3/19), 28.9% (11/38), 63.0% (17/27) and 100% (12/12) of the resultant mutant *Nfatc1^Cre^;Pik3ca^H1047R^* embryos died at E12.5, E13.5, E14.5 and E15.5, respectively (Table S3), indicating possible variations on the efficiency of PIK3CA^H1047R^ overexpression in ventricular EdCs of different embryos. We then switched to utilization of a recently developed strong bacterial artificial chromosome (BAC) transgenic line, in which Cre is inserted into exon 1 of the *Nfatc1* gene in the BAC construct (Fig. S8A), so expression of Cre is driven by *Nfatc1* regulatory elements. This new BAC *Nfatc1Cre* line (*Nfatc1^CreBAC^*) drives robust recombination specifically throughout the atrial and ventricular endocardium in addition to cushion endocardium and lymphatic ECs, but not in the ECs of the peripheral vasculature, including the branchial arch arteries, descending aorta and more distal pulmonary arteries (Fig. S8B-M). Thus, the expression pattern of this new *Nfatc1^CreBAC^* line faithfully recapitulates endogenous Nfatc1 expression and the knock-in *Nfatc1Cre* line (Wu et al., 2012; Zhou et al., 2015). Global *Tie2* knockout and endocardial deletion of *Tie2* via the knock-in *Nfatc1^Cre^* led to early embryonic lethality at E10.5 and E13.5, respectively (Dumont et al., 1994; Qu et al., 2019; Sato et al., 1995). Surprisingly, endocardial deletion of *Tie2* via the new *Nfatc1^CreBAC^* caused embryonic lethality of *Nfatc1^CreBAC^;Tie2^f/f^* right after E11.5 (Table S3; Fig. S9A), 2 days earlier than using the knock-in *Nfatc1^Cre^*. Their defects in endocardial network (simplified endocardial network) and trabeculation (fewer but thicker trabeculae) were more severe than those using the knock-in *Nfatc1^Cre^* line (Fig. 5A-C). Accordingly, immunostaining with anti-TIE2 antibody revealed almost complete loss of TIE2 in *Nfatc1^CreBac^;Tie2^f/f^* endocardium at E11.5 (Fig. S9B), while very low level of the mosaic TIE2 expression was observed in *Nfatc1^Cre^;Tie2^f/f^* endocardium (Qu et al., 2019), suggesting that there is a dosage-sensitive dependence of endocardial TIE2 for the severity of the phenotype. Similarly, endocardial-forced activation of FOXO1 via *Nfatc1^CreBac^* led to earlier embryonic lethality and more severe trabeculation defects than using the knock-in *Nfatc1^Cre^* line (Table S3; Fig. S9C,D). These results suggest that this new *Nfatc1^CreBAC^* line is more efficient than the knock-in *Nfatc1^Cre^* line.

When this new *Nfatc1^CreBAC^* line was crossed with female homozygous floxed *Pik3ca^H1047R^* mice, all (15/15) of the resultant mutant embryos (*Nfatc1^CreBac^;Pik3ca^H1047R^*) died right after E11.5 (Table S3; Fig. S9E), the same lethality time point as *Nfatc1^CreBAC^;Tie2^f/f^* (Fig. S9A), earlier than the above *Nfatc1^Cre^;Pik3ca^H1047R^* (Table S3), further supporting that this *Nfatc1^CreBAC^* line is more efficient than the knock-in *Nfatc1^Cre^* line. To understand the cause of lethality, *Nfatc1^CreBac^;Pik3ca^H1047R^* and control littermates were examined at E11.5 with immunostaining and Alcian Blue staining. This revealed reduced cardiac trabeculation (reduced total number of trabeculae, and ratio of overall trabecular area and length of the compact wall) in the mutants, compared with littermate controls, despite the presence of abundant EdCs and normal development of atrioventricular canal (AVC) and outflow tract (OFT) cushions (Fig. 5D-F). As expected, immunostaining of this heart at E11.5 for FOXO1 revealed that almost all EdCs displayed a diffuse nucleocytoplasmic localization of FOXO1 (Fig. S9F). These findings suggest that the PI3K signaling is directly involved in cardiac trabeculation and its abundance is finely modulated.

### scRNAseq reveals the ECM profiles of EdCs implicated in TIE2 signaling during trabeculation

**Fig. 6.**
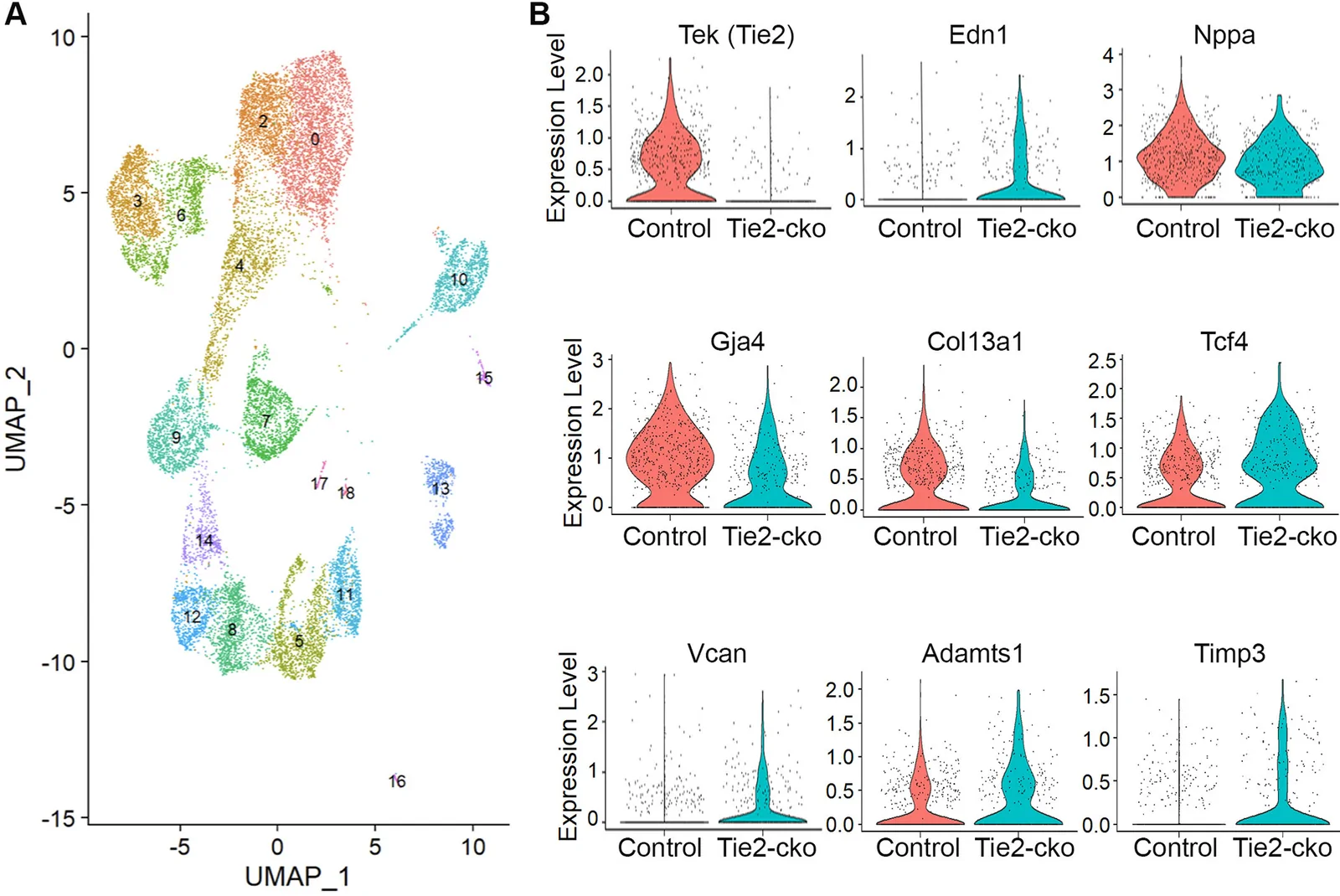
**Identification of versican metabolism as implicated in TIE2 signaling during trabeculation via scRNAseq.** (A) UMAP plot of all captured cell populations colored by cluster. (B) Violin plots showing alteration on expression of selected genes in E10.5 wild-type and *Tie2-cko* ventricular EdCs.

To identify additional targets of TIE2 signaling during trabeculation, we performed scRNAseq on *Tie2-cko* and littermate control hearts at E10.5. We obtained high-quality scRNAseq profiles for 9328 and 10,243 cells from *Tie2-cko* and control heart single cell suspensions, respectively. Unsupervised clustering revealed 18 clusters (Fig. 6A). Based on expression of lineage genes (de Soysa et al., 2019; DeLaughter et al., 2016; Jang et al., 2024; Li et al., 2016, 2019; Wu et al., 2022), it is apparent that each cluster represents a unique cell type. Clusters 0, 2, 3, 4 and 6 represent CMs, which highly express genes such as *Tnni3*, *Ttn* and *Myl1*. Clusters 5 and 11 are two endocardial EC clusters expressing *Cdh5*, *Tie1*, *Nrg1*, *Plxnd1*, *Ecscr* and *Pecam1*. Between these EC clusters, cluster 11 has a relatively higher expression of *Emcn*, *Npr3*, *Gata2* and *Gpr182* compared to cluster 5, suggesting it is comprised of atrial and ventricular ECs. Cluster 5, on the other hand, has higher *Nfatc1* expression than EC cluster 11, implying a cushion EC population. Clusters 8, 9, 12 and 14 are mesenchymal cells (MCs), which specifically express MC marker genes such as *Snai1* and *Fbln2*. Cluster 7 is an epicardial cell cluster, expressing *Upk1b* and *Upk3b*. Clusters 10, 16, 17 and 18 represent red blood cells or other non-cardiac cells. Together, the clusters covered all the cardiac cell types at E10.5 (de Soysa et al., 2019; DeLaughter et al., 2016; Li et al., 2016). Differential expression analysis revealed that *Tie2* was efficiently knocked down, *Cdkn1c* and Nppa were downregulated in both CMs and EdCs; two FOXO1 downregulated genes *Col13A1* and *Gja4* were downregulated, while *Edn1* was upregulated in EdCs of *Tie2-cko* (Fig. 6B; Table S4), consistent to our bulk RNAseq results (Qu et al., 2019). Interestingly, increased expression of additional FOXO1 upregulated genes (*Egr1*, *Tcf4*, *Pdgfb* and *Timp3*) (Daly et al., 2004; Korhonen et al., 2016; Papanicolaou et al., 2008; Potente et al., 2005) was observed in the mutant EdCs (Fig. 6B). Endocardial marker *Emcn* (endomucin) was also upregulated, consistent to more intense immune staining in the mutant EdCs than in the control (Figs 1,2; Fig. S2). However, *Tie2-cko* EdCs demonstrated normal expression of a list of EdC-specific genes [such as *Sox7*, *Arap3*, *Tmem100*, *Npr3*, *Nfatc1*, *Gpr126* (*Adgrg6*), *Rasip1*, *Kdr*, *Vwf*, *Cdh5* and *Gata2*] (Table S4), in contrast to their decreased expression previously seen in E11.5 *Tie2-cko* hearts via bulk RNAseq (Qu et al., 2019). This reduction of endocardial expression was likely secondary to dramatically reduced EdC number in the whole mutant hearts. Consistently, when *Sox7*, *Arap3* or *Tmem100* were deleted specifically from the endocardium using *Nfatc1^Cre^*, all the resultant mutants displayed a normal endocardial network and proper trabeculation (Qu et al., 2019; Hong et al., 2021).

Among the differentially expressed genes identified, we are particularly interested in the genes associated with ECM composition and metabolism, because the secretion of ECM from endocardial cells supports trabeculation and the normal dynamic expression of ECM proteins is essential for normal CM proliferation and trabeculation (Jang et al., 2024; Stankunas et al., 2008). Although our previous Alcian Blue staining suggested no alteration in total ECM or acellular cardiac jelly in *Tie2-cko* embryonic hearts (Qu et al., 2019), we found that the expression of a cadre of ECM-associated genes, including *Hbegf* (heparin-binding EGF-like growth factor), *Ptn*, *Tgfbi* (transforming growth factor, beta induced), *Col4a1*, *Col4a2*, *Adamts1*, *Ccdc80* (coiled-coil domain containing 80), *Itga1* (integrin alpha 1), *Sparc* (secreted acidic cysteine rich glycoprotein), *S100a6* (S100 calcium binding protein A6), *Vcan* and *Timp3* was highly enriched in the mutant cluster 11. However, the expression of other ECM-associated genes, including *Col13a1*, *Col3a1*, *Col26a1* and *Hapln1* (hyaluronan and proteoglycan link protein 1, an ECM component believed to stabilize hyaluronan–Vcan interactions; Wirrig et al., 2007), was significantly reduced in the mutant cluster 11 as compared to control (Fig. 6B; Table S4). In particular, *Vcan* expression was upregulated by 56.3% in *Tie2-cko* heart ECs (cluster 11) compared with control EdCs, while unchanged in cells of other clusters. Interestingly, the expression of *Adamts1* in *Tie2-cko* hearts was upregulated by 67.3%, while other Adamts genes (including *Adamts4*, *5*, *9* and *10*) remained unchanged compared with control cluster 11 EdCs. Surprisingly, the expression of *Timp3* was also upregulated by 108.3% in *Tie2-cko* cluster 11, while the other three Timp genes remained unchanged (Fig. 6B; Table S4). Thus, these results suggest that TIE2 signaling in endocardium may modulate trabeculation by regulating ECM composition and metabolism via dynamic abundance of ADAMTSs and TIMP3.

### Loss of *Tie2* in endocardium led to abnormal cardiac ECM composition and metabolism

**Fig. 7.**
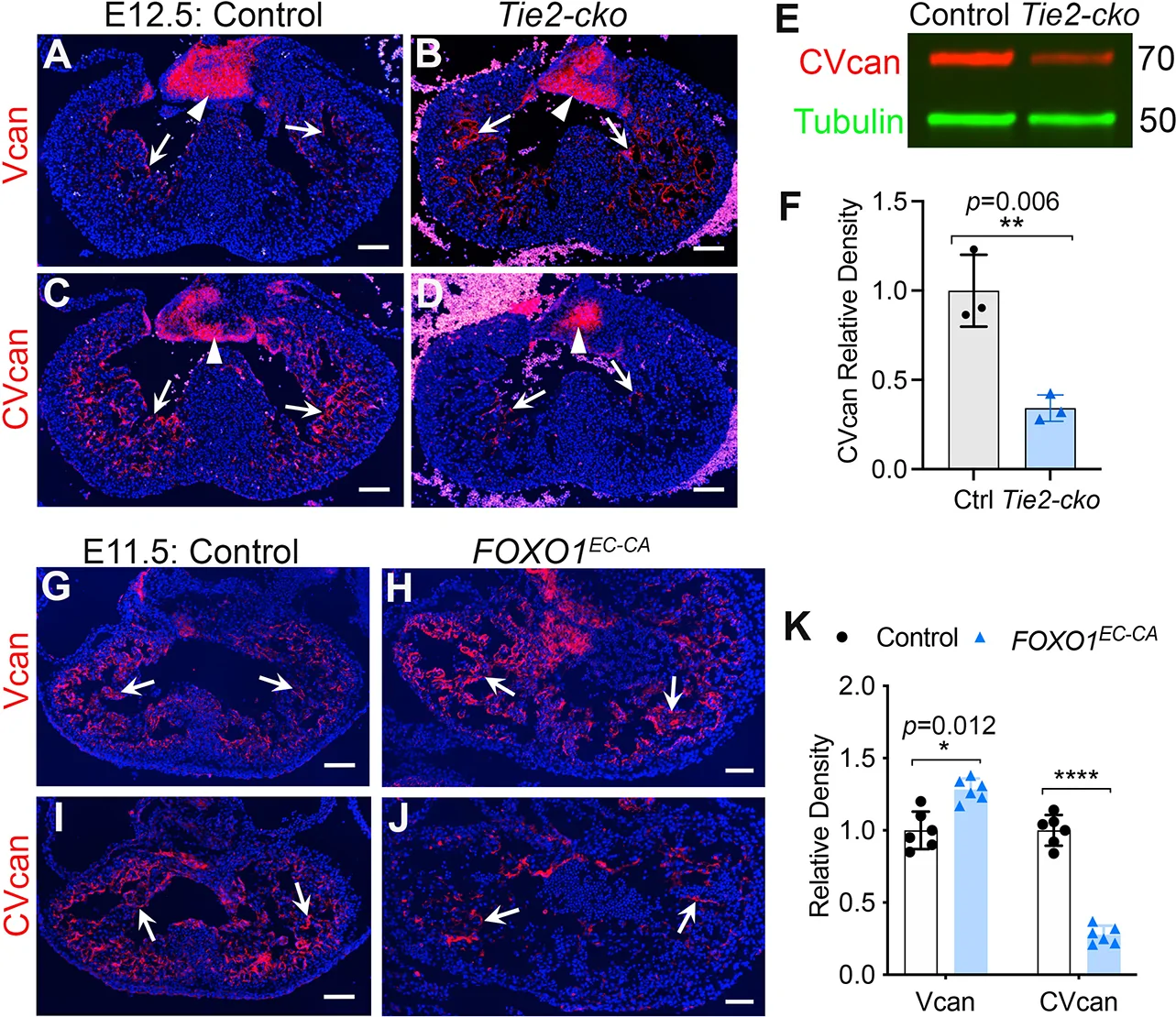
**Endocardial loss of *Tie2* and forced activation of FOXO1 resulted in enhanced expression of versican but impaired versican cleavage.** (A-D) Immunostaining of heart sections at E12.5 showing that the expression of intact versican (Vcan) was significantly increased, whereas cleaved Vcan (CVcan, DPEAAE) was dramatically reduced in *Tie2-cko* ventricles, compared to their littermate controls. (E,F) Images of representative immunoblot analysis (E) and comparison of CVcan (F) in whole lysate of the hearts from E12.5 wild-type and *Tie2-cko* embryos. *n*=3× two embryos per group. (G-K) Representative immunostaining images and quantitative analysis of E11.5 control and *FOXO1^EC-CA^* hearts showing enhanced Vcan expression and reduced CVcan expression in *FOXO1^EC-CA^* ventricles, compared to their littermate controls. Arrows show representative positive staining in ventricles; arrowheads show positive staining in AVC cushions. DAPI was used to counterstain nuclei in all images. Data represent the mean±s.e.m. Two-tailed unpaired Student's *t*-test; *P<0.05; **P<0.01; ****P<0.001. Scale bars: 100 μm.

Our scRNAseq analysis prompted us to re-evaluate the potential alteration of ECM in *Tie2-cko* embryonic hearts with immunostaining*.* In wild-type (WT) embryos during trabeculation, intact Vcan was highly expressed in the endocardial cushions and relatively weakly expressed in the area between the muscular ventricular trabeculae and EdC layer, sharing the similar distribution with cleaved versican (CVcan) (Fig. 7, Fig. S10). From the early stage to the later stages, Vcan expression around the trabeculae was gradually decreased. However, CVcan expression was significantly increased, indicating gradual cleavage of Vcan during trabeculation. In *Tie2-cko*, *Nfatc1^CreBAC^;Tie2^f/f^* and *FOXO1^EC-CA^* ventricles, Vcan expression around the trabeculae failed to decrease and CVcan expression failed to increase, suggesting impaired cleavage of Vcan over time (Fig. 7; Fig. S10A-L). Further western blot analysis confirmed that the total amount of Vcan in the whole heart was reduced by 65.8% in *Tie2-cko* embryos at E12.5 compared with control (Fig. 7E,F). No alteration in Vcan or CVcan expression was observed in endocardial cushions between *Tie2-cko* and littermate control embryonic hearts during trabeculation (Fig. 7A-D; Fig. S10A-H). Expression of other major components of the cardiac ECM including hyaluronan (Fig. S10M,N), fibronectin, laminin and heparan sulfate proteoglycan (Qu et al., 2019) was not altered. Thus, these results indicate that endocardial loss of TIE2 led to Vcan accumulation uniquely due to impaired Vcan cleavage, which resulted in abnormal proliferation of CMs during trabeculation.

**Fig. 8.**
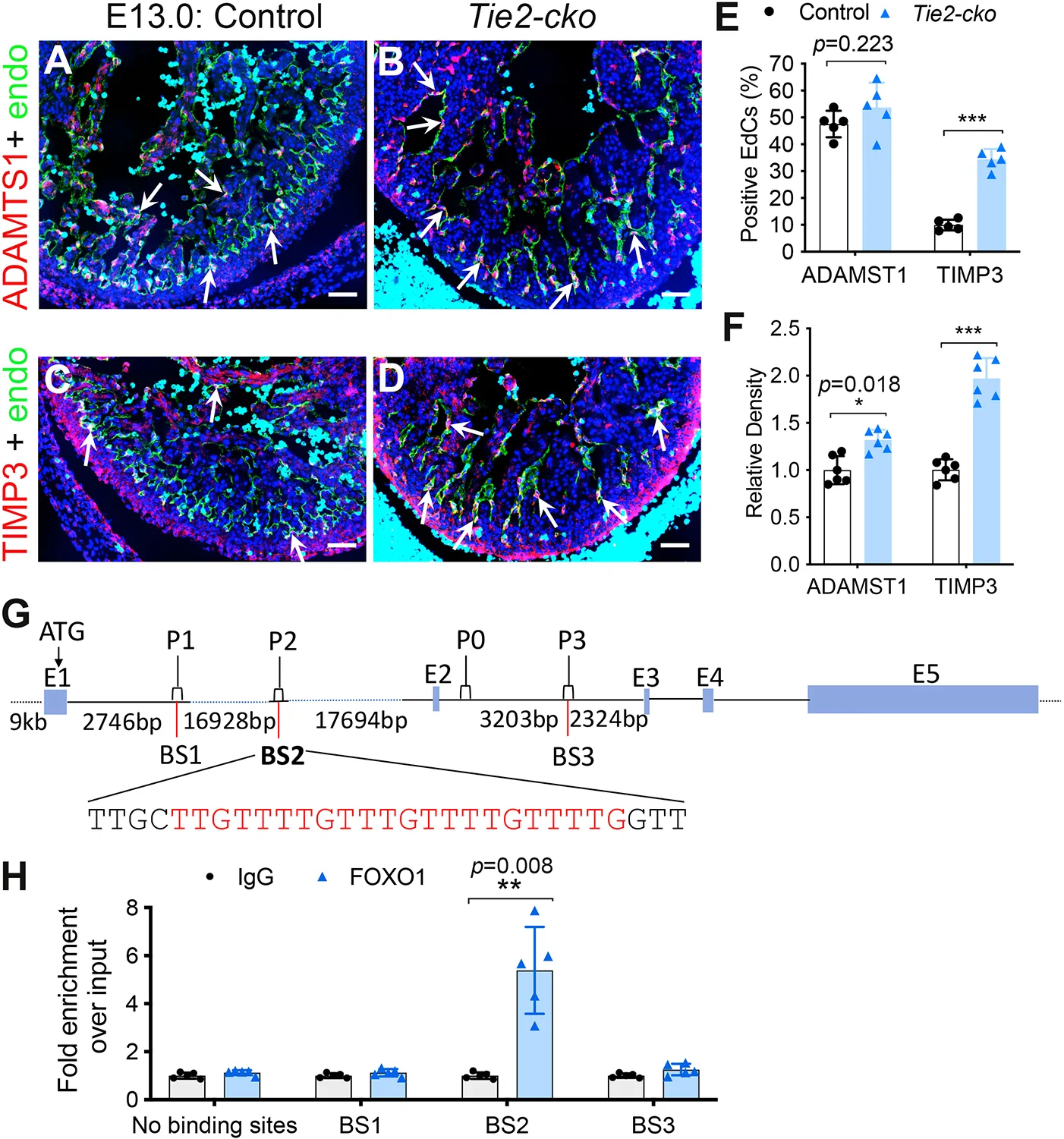
**Endocardial loss of *Tie2* resulted in enhanced expression of ADAMTS1 and TIMP3 in ventricular EdCs.** (A-D) Control and *Tie2-cko* ventricular sections at E13.0 were co-stained for endomucin (green) and ADAMTS1 (red; A,B) or TIMP3 (red; C,D). Arrows show representative positive staining in ventricular EdCs. DAPI was used to counterstain nuclei in all images. (E,F) Quantitative analysis of A-D showing that, compared to their littermate controls, relative staining density of ADAMTS1 was increased in *Tie2-cko* hearts, although no change was detected on the percentage of ADAMTS1^+^ EdCs. Relative staining density of TIMP3 and the percentage of TIMP3^+^ EdCs were, however, both increased in the mutant ventricles. (G) Schematic representation of mouse chromosome 10 region (corresponding to NCBI reference sequence NC_000076.7: base 86136201-86174350) indicating the position of the three putative FOXO1-binding sites (BS). Exonic regions are in blue and the ATG is located in exon 1. The four overlapped insulin response elements (IREs) in the second BS are in red. The primer pairs flanking the putative FOXO1-binding sites (BS1-3) and the pair flanking an intronic region between exon 2 and BS3 without FOXO1 binding sites (no binding site) are indicated as P1, P2, P3 and P0, respectively. (H) ChIP-qPCR showing significant enrichment (≈5-fold) of DNA/FOXO1 complexes only on BS2 within the *Timp3* locus following chromatin immunoprecipitation using IgG or an antibody specific to FOXO1. Data represent the mean±s.e.m. Two-tailed unpaired Student's *t*-test; *P<0.05; **P<0.01; ***P<0.001. Scale bars: 50 μm.

Next, we used immunostaining to assess expression of ADAMTS1 and TIMP3 in *Tie2-cko* ventricles. In WT embryos, ADAMTS1 was detected mainly in ventricular EdCs during trabeculation, and about half (47.5%) of all EdCs at E13.0 were positive for ADAMTS1 (Fig. 8A,E). Compared to these littermate controls, no change was observed in the percentage of ADAMTS1^+^ EdCs in *Tie2-cko* hearts, but the relative density of ADAMTS1 staining was significantly increased (Fig. 8B,E,F; Fig. S11A-D), even though their endocardial network was simplified due to loss of EdCs. Consistent to previous reports that *Timp3* mRNA is expressed in the developing endocardium and remodeling myocardium (Brauer and Cai, 2002), our immunostaining revealed that TIMP3 was weakly expressed in both ventricular EdCs and some CMs of WT embryonic heart (Fig. 8C,D; Fig. S11E,F). Compared to only 9.9% TIMP3^+^ EdCs in the control ventricles at E13.0, 34.4% of EdCs in the mutants were positive for TIMP3 staining. Consistently, the relative density of TIMP3 staining in the mutants increased by 97.2%, even though the number of EdCs was significantly reduced (Fig. 8C-F). Interestingly, endocardial forced activation of FOXO1 also led to enhanced expression of ADAMTS1 and TIMP3 during trabeculation (Fig. S12) in *Tie2-cko* embryos. Thus, the above results suggest that TIE2 signaling in endocardium may modulate trabeculation by regulating ECM composition and metabolism via preventing inhibition of ADAMTSs by excessive TIMP3.

To investigate if *Timp3* is a direct target of FOXO1, we performed an *in silico* analysis of the mouse *Timp3* locus and identified three potential FOXO1 binding sites in the gene body, two are located between exon 1 and exon 2 and the third is in the intron between exon 2 and exon 3. The first and the third binding site each contain a single consensus FOXO1 DNA binding sequence – insulin response element (IRE) TTGTTTAC (Brent et al., 2008) – while the second has four overlapped IREs (Fig. 8G). To determine whether FOXO1 binds to the three potential binding sites in *Timp3* genome, we performed ChIP-qPCR using the mouse cardiac ECs, which were used to obtain a sufficient number of cells for this assay. The result showed the binding of FOXO1 to the second binding site only, but not the first and the third (Fig. 8H). These observations suggest that *Timp3* is a direct target of FOXO1 in the developing endocardium that mediates the FOXO1-dependent ECM composition and metabolism.

## DISCUSSION

We previously reported that EdC-specific deletion of *Tie2* via the knock-in *Nfatc1^Cre^* led to embryonic lethality at E13.5 due to impaired endocardial growth and hyperplastic trabeculation in the mutant embryonic hearts (Qu et al., 2019). Deletion of *Tie2* via the new BAC *Nfatc1^Cre^* in the current study resulted in earlier lethality at E11.5 due to stronger cardiac phenotypes. Utilizing multiple genetic models, we provide additional insights into the mechanisms of TIE2 signaling during cardiac trabeculation. We find that endocardial deletion of *Tie2* resulted in increased nuclear localization of FOXO1, reduced phosphorylation of AKT and reduced expression of several proliferation promoting genes (including cyclin D1) in EdCs. The cardiac defects seen in EdC *Tie2-*deficient hearts were phenocopied by a FOXO1 gain-of-function model and partially rescued by loss of *Foxo1*. Furthermore, excessive PI3K signaling in EdCs caused reduced trabeculation. Moreover, our scRNAseq, immunostaining and western blot analyses on embryonic hearts revealed that endocardial loss of *Tie2* led to impaired cleavage of versican, an ECM protein promoting CM proliferation, which was associated with enhanced expression of ADAMTS endogenous inhibitor TIMP3 in *Tie2-cko* EdCs. Further, our ChIP assay suggests that *Timp3* is a direct target of FOXO1 in the developing endocardium.

During cardiac trabeculation, EdCs and CMs support and interact with each other to stimulate CM proliferation and endocardial growth (Qu et al., 2022). Even as studies continue to highlight the essential roles of the endocardium, the regulatory mechanisms involved in endocardial proliferation and development during cardiac trabeculation remain largely unknown. Interestingly, despite the observation that the early endocardium (E7.5) uniquely expresses NFATC1, the transcription factor is not necessary for regulating the specification or proliferation of EdCs, although it is later necessary for the formation of cardiac valves (Ranger et al., 1998; Harris and Black, 2010; Wu et al., 2011). ETV2 and VEGFR2 are both specifically expressed in ECs (including EdCs) and are essential for their specification, because loss of either gene results in embryonic mortality with global depletion of all ECs (Ferdous et al., 2009; Milgrom-Hoffman et al., 2011; Shalaby et al., 1995). However, conditional knockout mice of *Etv2* with either *Tie2^Cre^* (active in all ECs) or *Nfatc1^Cre^* (active in EdCs from E9.0, about 1 day later than *Tie2^Cre^*) did not show abnormal endocardium (Park et al., 2016; Del Monte-Nieto et al., 2018; Qu et al., 2019). Similarly, endocardial-specific deletion of *Vegfr2* with *Nfatc1^Cre^* also led to no apparent endocardial defects (Fig. 4), although most null embryos died between E16.5 and E18.5 with abnormalities in coronary angiogenesis and myocardial vessel formation (Wu et al., 2012). Thus, endocardial specification is ETV2- and VEGFR2- dependent, whereas the subsequent endocardial growth and expansion during trabeculation are not.

It is evident that TIE2-ANG1 and Notch1-Dll4 signaling pathways are both required for endocardial growth during trabeculation, as endocardial-specific deletion of *Tie2* (or myocardial deletion of *Ang1*) and *Notch1*, or its ligand *Dll4*, with *Nfatc1^Cre^* led to impaired endocardial sprouting and a simplified endocardial network (D'Amato et al., 2016; Del Monte-Nieto et al., 2018; Kim et al., 2018; Qu et al., 2019). While the mechanism(s) by which Notch1-Dll4 signaling modulates endocardial proliferation during the critical trabeculation timeframe and its potential interactions with TIE2-ANG1 signaling remain to be explored, our results reveal that FOXO1 is a direct target of TIE2 activation in the endocardium.

FOXO1, a transcription factor, exerts most of its biological control in the nucleus, where it acts alone or in complexes to control both histone remodeling and gene expression (Hatta and Cirillo, 2007; Huang and Tindall, 2007; Brent et al., 2008). It is a suppressor of both endothelial growth and proliferation in vascular ECs (Wilhelm et al., 2016; Menghini et al., 2020). When phosphorylated at three conserved sites by Akt, FoxO1 is exported from the nucleus, whereas unphosphorylated FOXO1 resides in the nucleus. Therefore, its transcriptional activity is negatively regulated by Akt, and its subcellular localization is dynamically regulated by mitogenic signaling (such as ANG1, insulin/insulin-like growth factor 1, tumor necrosis factor 1, platelet-derived growth factor and angiotensin II) and various extracellular stimuli (such as shear stress and serum deprivation) in vascular ECs (Eklund et al., 2017). FOXO1 is highly co-expressed with TIE2 in all ECs including EdCs and lymphatic ECs. It is more localized primarily in the cytoplasm in vascular ECs and embryonic EdCs, in contrast to more nuclear localization in lymphatic ECs (Baldwin et al., 2025). We demonstrated that *Tie2* deficiency altered subcellular localization of FOXO1 in the endocardium. In agreement with increased nuclear localized of FOXO1 in the mutant EdCs, there was a dramatic reduction in phosphorylation of AKT, the regulator upstream of FOXO1 but downstream of TIE2 signaling in vascular ECs (Augustin et al., 2009; Eklund et al., 2017). Expression of a group of FOXO1 downregulated genes was likewise decreased, while expression of a cadre of FOXO1 upregulated genes was increased. In addition, impaired proliferation of EdCs was associated with reduction in expression of several proliferation promoting genes (including cyclin D1, *cMYC*) and p*YAP* in the mutant EdCs. PI3K is regulated by almost all receptor tyrosine kinases, including TIE2 and VEGFR2, and the two well-studied effectors of the PI3K pathway are AKT and mTOR (mammalian target of rapamycin) (Fruman and Rommel, 2014). Utilizing a gain-of-function model and the strong BAC *Nfatc1^Cre^* line, we show that excessive PI3K signaling in EdCs led to early embryonic lethality due to reduced cardiac trabeculation. Although VEGF/PI3K/AKT signaling has also been reported to promote FOXO1 cytoplasmic localization in a context-dependent manner, resulting in its inactivation in ECs *in vitro* (Abid et al., 2004; Kim et al., 2019), our endocardial-specific deletion of *Vegfr2* via *Nfatc1^Cre^* did not lead to FOXO1 nuclear localization in EdCs, or any defects in myocardial trabeculation and endocardial growth, suggesting that endocardial FOXO1 nuclear localization *in vivo* is not dependent on VEGF-VEGFR2 signaling and TIE2 modulation of subcellular localization of FOXO1 in the endocardium is unique. Our findings suggest that the cardiac defects seen in *Tie2-cko* mice were largely phenocopied by forced activation of *FOXO1* mutants (*FOXO1^EC-CA^*) via *Nfatc1^Cre^*. The same alterations associated with impaired endocardial growth and hyperplastic trabeculation were observed, including reduced EdC proliferation and cleavage of Vcan in both *Tie2-cko* and *FOXO1^EC-CA^* hearts. These data strongly suggest that FOXO1 is a primary effector of TIE2 activation during trabeculation.

Vcan, a chondroitin sulfate proteoglycan, promotes CM proliferation, while Vcan cleavage by ADAMTS proteases inhibits cardiomyocyte proliferation during myocardial trabeculation (Cooley et al., 2012; Yamamura et al., 1997; Mjaatvedt et al., 1998; Nandadasa et al., 2014). Vcan proteolysis is finely modulated by ADAMTSs (Edwards et al., 2008; Stankunas et al., 2008; Nandadasa et al., 2014) and the tissue inhibitors of metalloproteases (TIMPs). ADAMTS1 is a major ADAMTS protease in endocardium and vascular ECs and is mainly secreted by the endocardium into cardiac jelly (Kern et al., 2006; Stankunas et al., 2008; Hatipoglu et al., 2009). Among the four mammalian TIMPs, TIMP3 is the master endogenous inhibitor to ADAMTSs (Edwards et al., 2008; Arpino et al., 2015; Spanò and Spanò, 2022). In *Tie2-cko* hearts, some ECM genes were downregulated and some were upregulated, while others remained unchanged compared with control. As in a previous report on a myocardial *Angpt1*-deficient model (Kim et al., 2018), we identified excessive Vcan accumulation and impaired Vcan proteolysis in *Tie2-cko* and *FOXO1^EC-CA^* embryonic hearts. However, in contrast to their finding that mRNA expression of *Adamts1*, *Adamts4*, *Adamts5* and *Adamts9* was all reduced but *Timp3* remained unchanged in the hearts of myocardial *Angpt1*-deficient embryos at E10.5 via RT-PCR, we did not observe any reduction of expression on these Adamts genes in our *Tie2-cko* hearts. On the contrary, we detected increased expression of both *Adamts1* and *Timp3* in *Tie2-cko* EdCs via scRNAseq, which was further confirmed by immunostaining: more intense staining of ADAMTS1 and TIMP3 was documented in EdC, even though the total number of EdCs in the mutants were reduced. To further validate that TIMP3 is a direct target of FOXO1 in ECs, our ChIP assay using a cardiac EC line showed that FOXO1 directly binds a transcriptional enhancer region located between exon 1 and exon 2 of mouse *Timp3* gene, which contains four overlapped FOXO1 binding motifs. These data are consistent with a previous report on human decidualization implicating FOXO1 in the regulation of genes involved in ECM remodeling, including *TIMP3* (Grinius et al., 2006). Because increased TIMP3 levels result in ECM accumulation, whereas loss of TIMPs leads to enhanced matrix proteolysis, we postulate that enhanced inhibition of ADAMTSs via increased TIMP3 impaired cleavage of Vcan in *Tie2-cko* hearts.

While vascular ECs and cardiac EdCs both utilize TIE2-FOXO1 axis during development, our data support that EdCs employ distinct responses from vascular ECs, including ECM regulation. Because cardiac defects in *Tie2-cko* embryos were only partially rescued by simultaneous endocardial deletion of *Foxo1* via *Nfatc1^Cre^*, a large part of the paracrine effect of TIE2 on CMs was not rescued. Since cMYC is a critical effector of FOXO1 in coordinating angiogenic EC metabolism and growth (Wilhelm et al., 2016; Kim et al., 2019), we initially hypothesized that cMYC is a primary target of the TIE2-FOXO1 signaling axis governing EdC proliferation. Surprisingly, the cardiac defects seen in *Tie2-cko* mutant mice could not be rescued by restoring cMyc signaling with *Nfatc1^Cre^*-mediated *cMYC* overexpression. Additionally, pan-EC deletion of *Foxo1* with *Tie2^Cre^* results in embryonic lethality due to defective vascular development (Furuyama et al., 2004; Hosaka et al., 2004; Wilhelm et al., 2016), while EdC deletion of *Foxo1* via *Nfatc1^Cre^* has no cardiac phenotype, further suggesting that there is a unique requirement for FOXO1 in endocardial growth compared to vascular development.

**Fig. 9.**
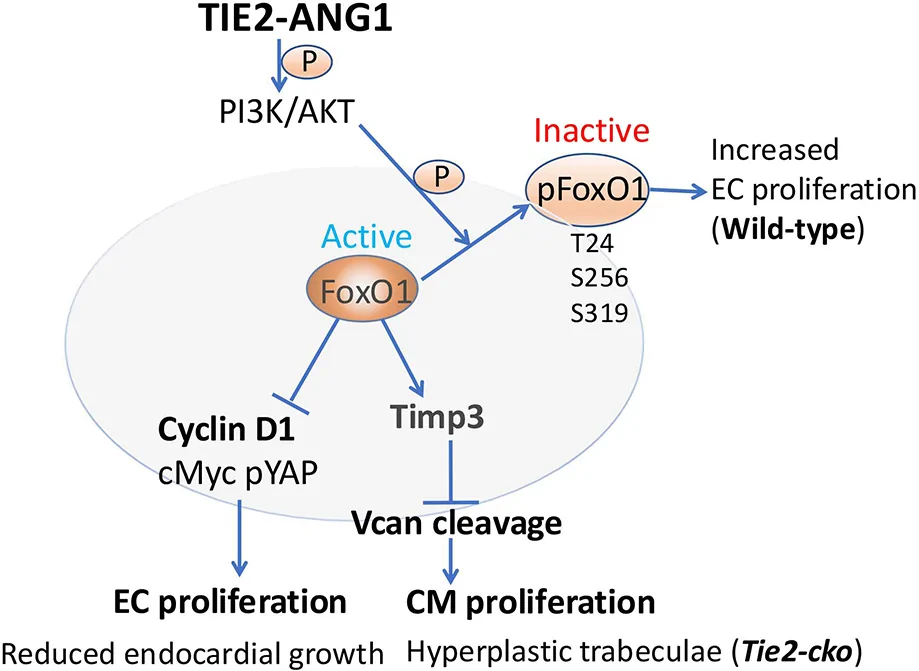
**Proposed TIE2 signaling pathway governing endocardial growth and CM proliferation during trabeculation.** In wild-type embryonic hearts, stimulation of TIE2 with ANG1 leads to the activation of PI3K/Akt, which in turn directly phosphorylates FOXO1 at three conserved residues (T24, S256 and S319), promoting its export from the nucleus by binding with the chaperone 14-3-3, and thus resulting in the inhibition of FOXO1-dependent transcription and normal proliferation of EdCs. In *Tie2-cko* and *FOXO1^EC-CA^* embryonic hearts, un-phosphorylated FOXO1 translocates to the nucleus, leading to induction of cell cycle withdrawal and inhibition of EdC proliferation, and increased TIMP3 production in EdCs. The latter impairs Vcan proteolysis by inhibition of ADAMTS1, which results in abnormally high CM proliferation and hyperplastic trabeculation.

In summary, we postulate that the primary autocrine function of EdC TIE2 signaling is to govern endocardial growth by uniquely sequestering FOXO1 within the cytoplasm via PI3K/AKT-mediated phosphorylation in EdCs (Fig. 9). This exclusion of FOXO1 from the nucleus promotes EdC proliferation and survival, which is essential for supporting myocardial trabeculation. One of the primary paracrine functions of TIE2 is to govern CM proliferation during trabeculation, promoting Vcan cleavage potentially via FOXO1-*Timp3*. This study provides a model for understanding the unique role of TIE2 signaling in the heart.

## MATERIALS AND METHODS

### Mice

The floxed mice *Tie2^f/f^* (Qu et al., 2019), *FOXO1^CA^* (a gift from Dr Ming O. Li at Memorial Sloan Kettering Cancer Center, New York, USA; Luo et al., 2016; Ouyang et al., 2012), *Vegfr2^f/f^* (a gift from Janet Rossant at Department of Molecular Genetics, University of Toronto, Canada; Albuquerque et al., 2009) and the constitutive knock-in *Nfatc1^Cre^* line (Wu et al., 2012) have been described previously. The floxed c*Myc^OE^* (#020458, Sander et al., 2012) and *Foxo1^f/f^* (#024756, Paik et al., 2007), *R26-Pik3ca^H1047R^* (#016977, Adams et al., 2011) and *R26-eGFP* (#010701) strains were purchased from the Jackson Laboratory. To generate an EdC-specific Cre transgenic mouse line, a cassette consisting of the *Cre* followed by dTomato and SV40 polyadenylation and a module containing the Kana selection marker flanked by frt sites was introduced into the coding ATG of the mouse *Nfatc1* gene carried by a BAC by VectorBuilder, Cyagen. The Kana cassette was deleted after Flp-mediated recombination (Fig. S7B). Transgenic offspring were analyzed for BAC insertion by genomic PCR amplification and end-sequencing. To verify their activity, these *BacNfatc1^Cre^* mice were mated with a reporter line (*R26-eGFP*). Cotransgenic progeny from these matings were analyzed by cryosection immunostaining.

All strains were maintained on a mixed 129 and C57/BL6 background and both sexes were used. To avoid recombination in the female germline, only *Cre*^+^ male mice were used for intercrossing. Genotyping was performed from embryonic yolk sacs (in the lab) or tail/ear samples (Transnetyx). For BrdU incorporation, pregnant females were injected intraperitoneally (i.p.) with BrdU cell proliferation kit (RPN20, Amersham Biosciences) at a dosage of 1 ml per 100 gram body weight 1.5 h prior to sacrifice. The animals were handled in accordance with institutional guidelines with the approval of the Institutional Animal Care and Use Committee of Vanderbilt University School of Medicine.

### Histological analyses and immunohistochemistry

For Alcian Blue staining, embryos were fixed in 4% paraformaldehyde (PFA) overnight, then dehydrated and embedded in paraffin, and cut into 6 μm sections. The preparation of paraffin and frozen sections and immunostaining were carried out following standard protocol as described previously (Qu et al., 2019). For paraffin sections, antigen retrieval was performed by boiling slides in 10 mM of citric acid buffer (pH 6.0) for 15 min before staining. Paraffin (6 μm) or frozen (10 μm) sections of mouse embryos were incubated overnight with primary antibodies (Table S1), followed by 1h incubation with a fluorescent-dye-conjugated secondary antibody. Alexa Fluor 488, 555, 594 and 647 fluorochrome-conjugated secondary antibodies (Invitrogen, A11055, A21432, A21447, A21206, A32572, A21244, A21208; 1:300) were used for signal detection. MYC, ADAMTS1, TIMP3, cyclin D1, phospho-YAP and phospho-Akt staining was enhanced using a tyramide amplification kit (TSA) coupled to Cy3 (1:100; NEL744, Perkin Elmer) or fluorescein (1:100, NEL741, Perkin Elmer). Hyaluronan was stained using biotinylated-hyaluronan binding protein, then developed with Alexa Fluor™ 555 streptavidin (1:300, S21381, Invitrogen). Images were visualized using an Olympus BX63 microscope and captured using a DP74 camera with cellSens Dimension image software (Olympus). Microscopic images are representative of three or more independent experiments. Microscopic image processing and analysis were performed using Adobe Photoshop software. For semi-quantitative analysis, the area and integrated optical density of the area were measured with tan expression using Image-Pro Plus 6.0 software, and the mean optical density was calculated.

### Morphometric analyses

The extent of the trabeculation defect was quantified by morphometric measurements of the number of trabeculae, the thickness of individual trabeculae and the compact zone, and total trabecular area as described (Chen et al., 2009; Qu et al., 2019). The total degree of trabecular atrophy was measured by normalizing trabecular area to compact wall length. Transverse heart sections of control and mutant embryos were stained with anti-endomucin (endocardial marker) and anti-Tnnt2 (recognizes myocardial troponin T) to facilitate visualization of ventricular structures. For the thickness of the compact myocardium, the left ventricle was measured. ImageJ was used for the measurements.

### Quantification of incorporated BrdU and other immunostainings

Cell proliferation was analyzed by detection of BrdU incorporation and immunostaining for Ki67. For BrdU, Ki67, pAkt, cyclin D1, cMyc, pYAP, ADAMTS1 and TIMP3 quantification, the number of EdCs positive for endomucin or ERG was divided by the total number of EdCs, counted on at least four sections in each of five embryos of each genotype. All imaging quantifications (including relative intensity) were carried out using ImageJ software.

### Western blotting

Lysates from E12.5 embryos of the indicated genotypes, processed as previously described (Qu et al., 2019). A rabbit anti-versican V0, V1 Neo (DPEAAE) primary antibody was used to detect CVcan levels followed by a goat anti-rabbit StarBright™ Blue 700 (1:2500; Bio-Rad, 12004161) secondary antibody (Table S1). For protein normalization, membranes were simultaneously probed with a directly conjugated human anti-Tubulin hFAB™ Rhodamine antibody. All images were captured using the ChemiDoc™ MP Imaging System and analyzed with Image Lab Software.

### Quantitative RT-PCR

As previously described (Qu et al., 2019), briefly, control and *Tie2-cko* mutant embryos at E11.5 were dissected in ice-cold PBS. Whole hearts were separated from the rest of the body and RNA was extracted. Total RNA was purified using TRIZOL Reagent (Invitrogen) with additional DNase treatment (Promega). cDNA was then generated using the SuperScript III Reverse Transcriptase kit (Invitrogen). qPCR was performed on the CFX96 TouchTM thermal cycler (Bio-Rad) using iQTM SYBR Green Supermix (Bio-Rad). All assays were repeated at least twice, and all samples were run in triplicate. Analysis of gene expression was carried out using the comparative Ct (ΔΔCt) method as described by the manufacturer. Relative quantification of gene expression was normalized to *18S* mRNA expression level. Sequences of the PCR primers used are listed in Table S2.

### Chromatin immunoprecipitation assay

As a surrogate for endocardial ECs, we used immortalized mouse cardiac endothelial cells (MCECs; Cedarlane, CLU510) to perform chromatin immunoprecipitation (ChIP) analysis. These cells were prepared from microvascular neonatal MCECs by transfection with lentiviral vectors carrying the SV40T antigen and human telomerase (Lu et al., 2022). The ChIP assay was carried out using the iDeal ChIP-qPCR Kit (Diagenode) according to the manufacturer's protocol. For each experiment, ∼2.5×10^7^ cells were used to generate the sonicated chromatin fragments at the range of 100-300 bp. Rabbit anti-FOXO1 (Cell Signaling Technology) and IgG (negative control) from the kit were used to detect FOXO1 specific binding of chromatin (Table S1). qPCR was performed as above with primers listed in Table S2. The primer pair P0 flanking an intronic region without FOXO1 binding sites were used as a negative control. Relative quantities of each chromatin bound fragment were normalized relative to the amount of input DNA.

### Single-cell RNA sequencing

For generation of scRNAseq datasets, the entire cardiogenic region was dissected at E10.5. Isolated hearts were dissociated as previously described (de Soysa et al., 2019) for analysis at the Vanderbilt Technologies for Advanced Genomics core. Samples with viability >90% were submitted for library preparation. Analysis was carried out using 10x Genomics Chromium Controller, a single-cell profiling technology enabling the analysis of large cell numbers at a capture efficiency of up to 65%. Around 10,000 cells of each genotype were encapsulated and libraries were prepared using 10x catalog Part Number 1000006, 1000080 and 1000020 following the manufacturer's protocol. The libraries were sequenced using the NovaSeq 6000 with 150 bp paired end reads. Somite-matched control and *Tie2* mutant replicate libraries from the same litter were pooled and sequenced in the same lane. All libraries were sequenced to a mean read depth of at least 50,000 total aligned reads per cell. Two hearts at each stage (for each genotype) from the same litter were analyzed. RTA (version 2.4.11; Illumina) was used for base calling and analysis was completed using 10x Genomics Cell Ranger software v2.1.1.

For analysis of scRNAseq datasets, Seurat (Butler et al., 2018) for R was used to normalize data, find highly variable genes and perform dimension reduction/clustering, and cell populations were defined based on cluster markers. sc-UniFrac (Liu et al., 2018a; Han et al., 2018) was used to statistically define proportional transcriptional shifts in CMs, EdCs and other populations in response to *Tie2* deletion by VANGARD (Vanderbilt Technologies for Advanced Genomics Analysis and Research Design).

### Statistical analysis

All experiments were repeated at least three times. Representative images are shown from at least five embryos (three independent litters)/genotype. All data are represented as mean±s.e.m. A two-sided Student's *t*-test was used to determine significant differences when comparing two experimental groups. One-way or two-way ANOVA with Tukey's multiple comparisons test was performed to test for significant differences when comparing multiple groups. A *P*-value of less than 0.05 was considered statistically significant. Graphpad Prism software (version 10) was used for all statistical analyses and to plot quantitative data.

## Supplementary Material



10.1242/develop.205674_sup1 Supplementary information
