# Supplementary material for: Endocardial TIE2 orchestrates cardiac trabeculation via sequestering FOXO1 within the cytoplasm and stimulating versican cleavage
Source: Development. 2026 Jul 29;153(14):dev205674. doi: 10.1242/dev.205674 (PMC13450898; doi:10.1242/dev.205674)
Supplement: Supplementary file 1 [file develop-153-205674-s1.pdf]

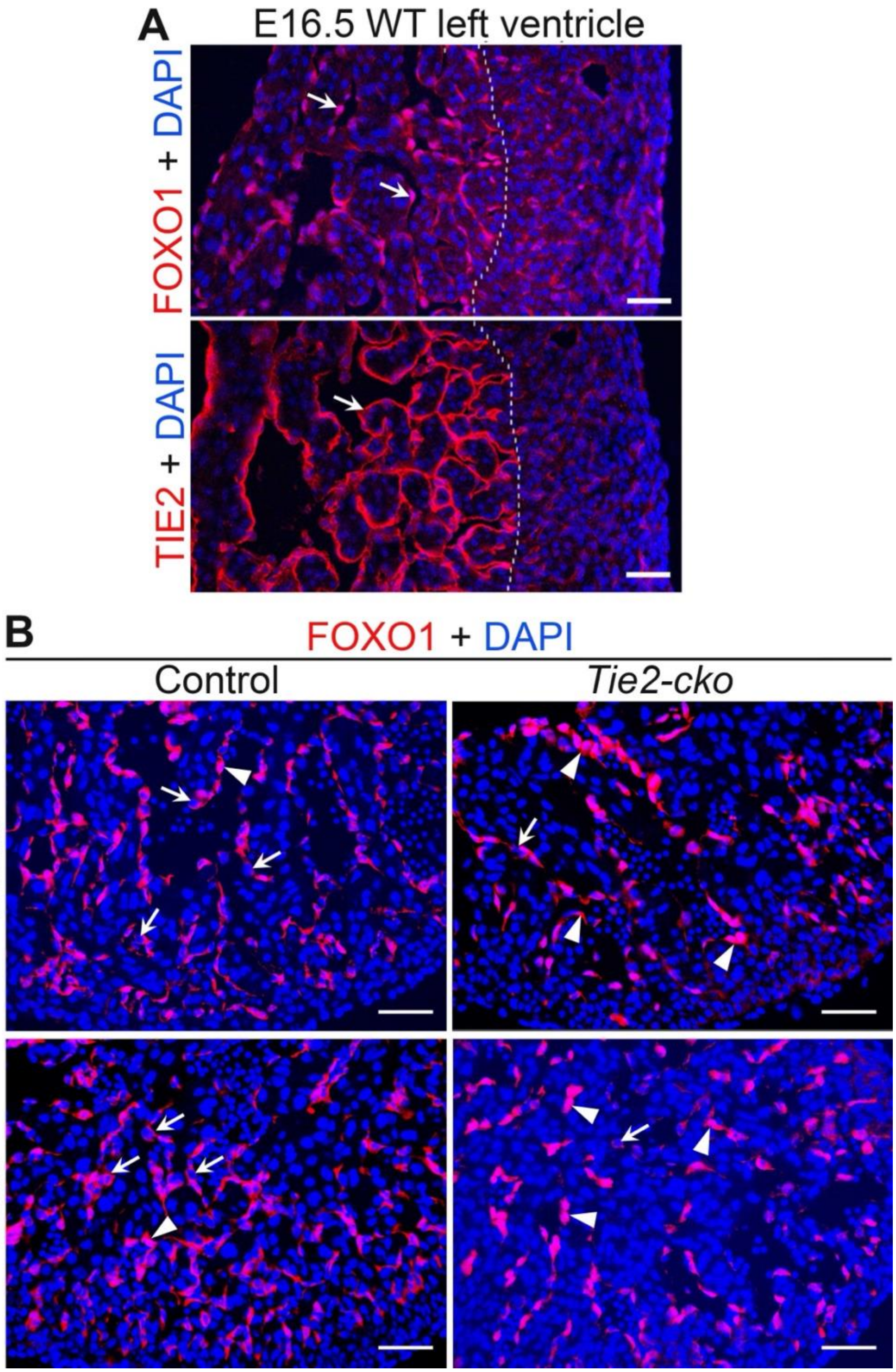

Development • Supplementary information

Fig. S1. FOXO1 is co-localized with TIE2 in the mouse embryonic heart and its cellular localization in EdCs is regulated by TIE2. (A) WT left ventricular sections at E16.5 were stained with FOXO1 and TIE2 antibodies respectively, showing that FOXO1 is highly co-localized and has the same expression pattern as TIE2 in the mouse embryonic heart. Both FOXO1 and TIE2 have much higher expression in the endocardium (left side of the dash line, arrow) than in coronary vessels (right side of the dash line). Note that most EdCs in WT at E16.5 demonstrated FOXO1 localized at nucleus (arrows). (B) Control (*Tie2*$^{+/f}$ and *Tie2*$^{f/f}$) and *Tie2-cko* left ventricular sections at E12.5 and E13.5 were stained with FOXO1 antibody, showing a diffuse nucleocytoplasmic localization (arrows) of FOXO1 in control ECs, but accentuated nuclear localization (arrowheads) in the mutant. DAPI was used to counter stain nucleus in all images. Scale bars: 50 µm.

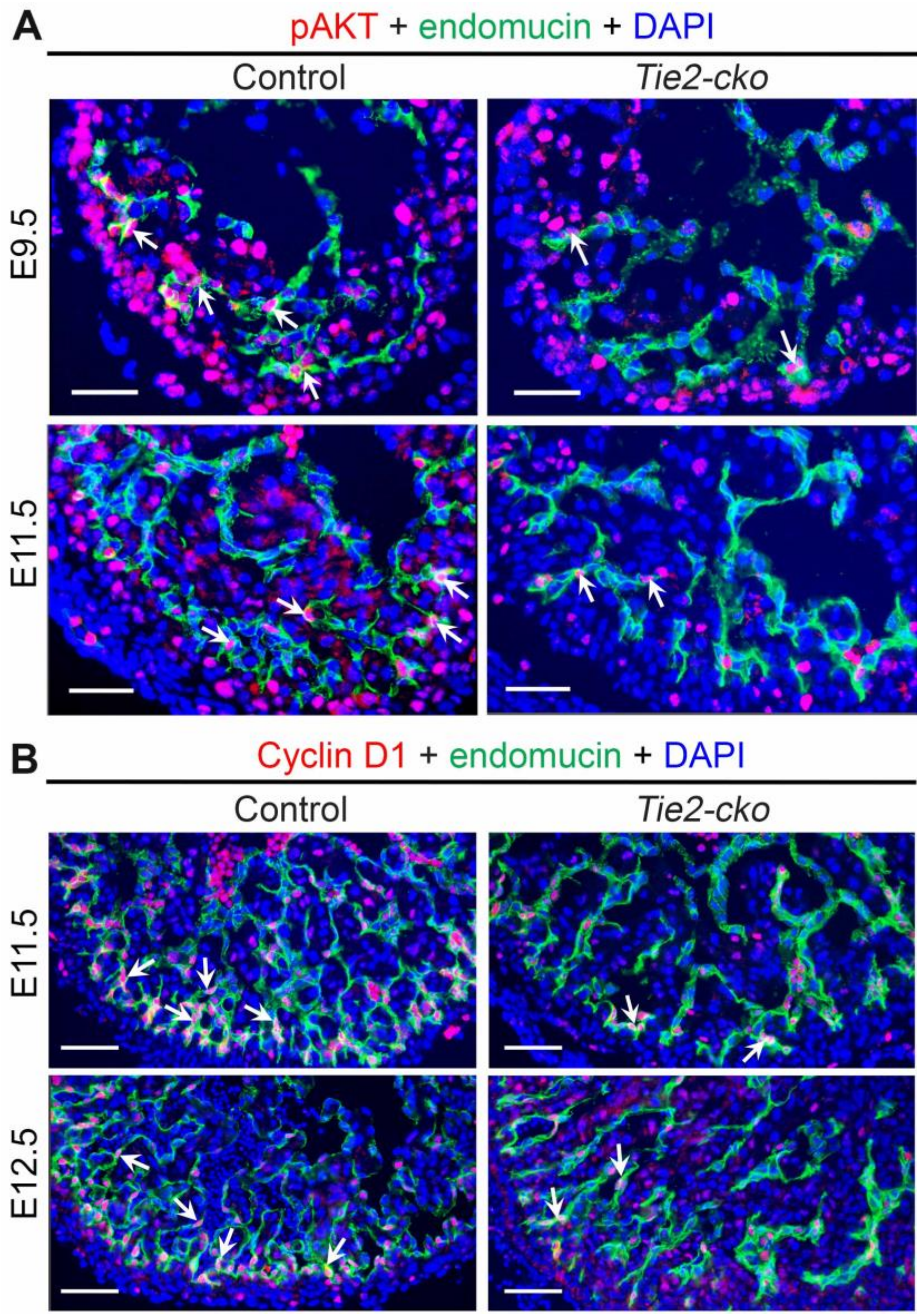

Fig. S2. Endocardial loss of *Tie2* resulted in reduced phosphorylation of AKT and reduced expression of Cyclin D1. (A) Control and *Tie2-cko* ventricular sections at E9.5 and E11.5 were co-stained with pAKT (red) and endomucin (green) antibodies. (B) Control and *Tie2-cko* ventricular sections at E11.5 and E12.5 were co-stained with Cyclin D1 (red) and endomucin (green) antibodies. Arrows, representative positive staining in EdCs. DAPI was used to counter stain nucleus in all images. Scale bars: 50 μm.

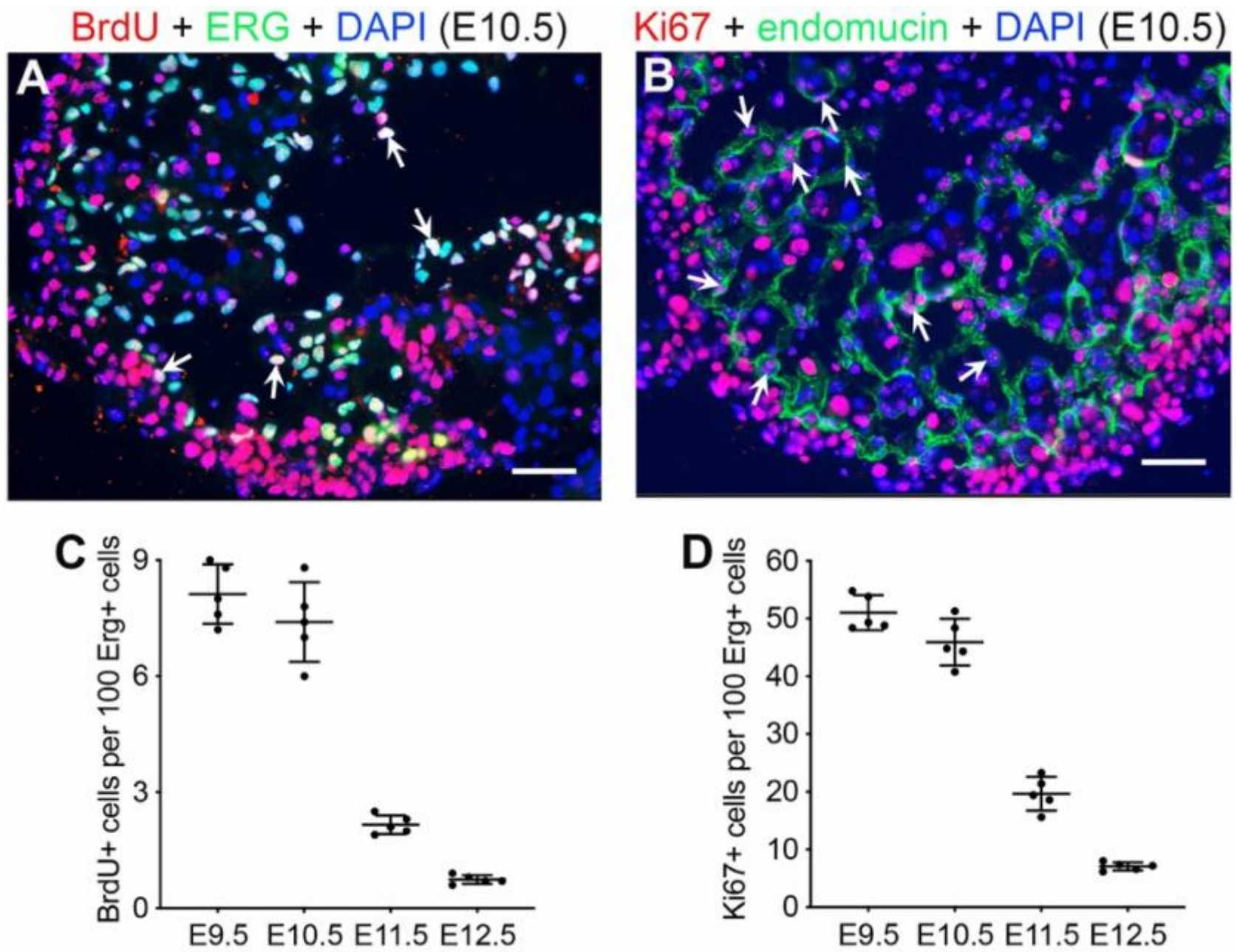

Fig. S3. EdC proliferation rate is decreasing with age during trabeculation. (A,B) WT ventricular sections at E10.5 were co-stained for BrdU/ERG (A) and Ki67/endomucin (B). Arrows, representative proliferating EdCs. (C,D) Quantification of BrdU or Ki67-positive cells as a percentage of total EdCs in endocardium. DAPI was used to counter stain nucleus in all images. Scale bars: 50 μm.

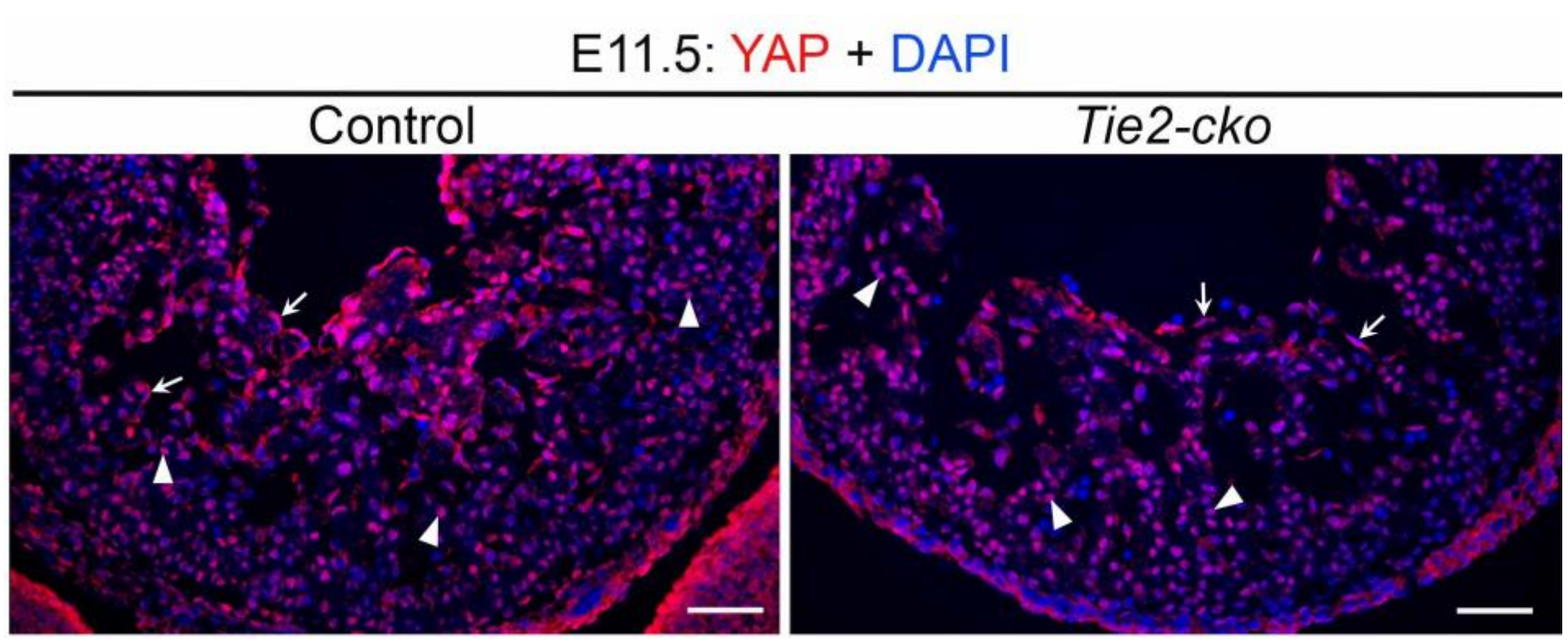

Fig. S4. Endocardial loss of *Tie2* led to no significant changes on YAP expression. Control and *Tie2-cko* left ventricular sections at E11.5 were stained with antibody against YAP. Arrows and arrowheads: representative positive staining in EdCs and CMs, respectively. DAPI was used to counter stain nucleus in all images. Scale bars: 50 μm.

Development • Supplementary information

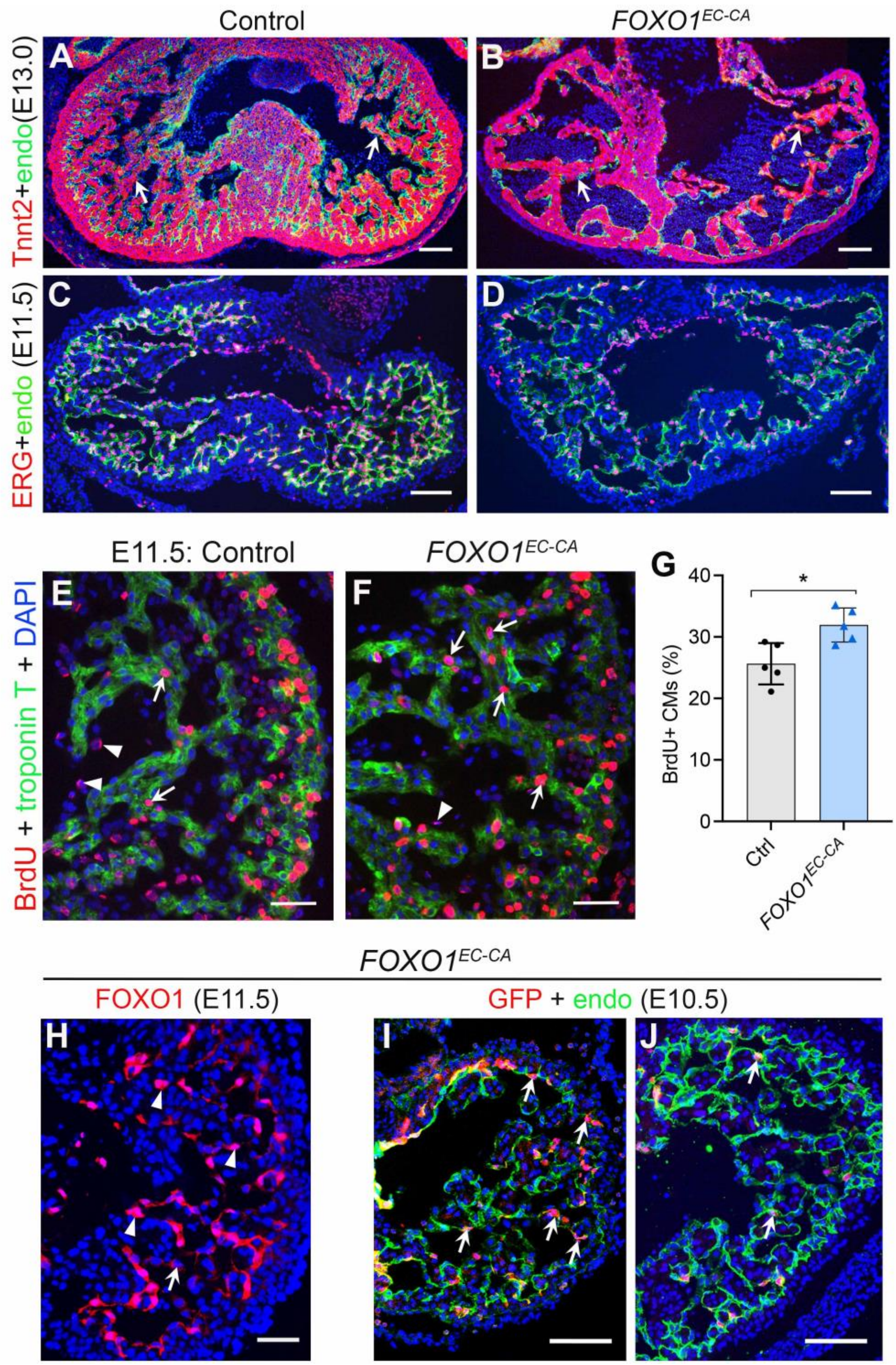
Control
FOXO1EC-CA
Tnnt2+endo(E13.0)
A
B
C
D
ERG+endo (E11.5)
E11.5: Control
FOXO1EC-CA
E
F
G
BrdU + troponin T + DAPI
BrdU+ CMs (%)
40
30
20
10
0
*
Ctrl
FOXO1EC-CA
FOXO1EC-CA
FOXO1 (E11.5)
GFP + endo (E10.5)
H
I
J

Fig. S5. Cardiac defects in *Tie2-cko* mice were phenocopied by endocardial forced activation of FOXO1. (A,B) Control (*FOXO1*$^{CA/+}$) and *FOXO1*$^{EC\text{-}CA}$ ventricular sections at E13.0 were stained for troponin T (Tnnt2, red) and endomucin (endo, green), showing abnormal trabeculae (arrows) and simplified endocardial network in the mutant heart. (C,D) Co-immunostaining of ventricular sections with ERG (red) and endomucin showing fewer ERG/endomucin-positive endocardial cells in the *FOXO1*$^{EC\text{-}CA}$ embryos at E11.5 than in the control. (E, F) BrdU pulse labeling and co-immunostaining with BrdU (red) and troponin T (green) showing more BrdU-positive cardiomyocytes (CMs, arrows) but fewer BrdU-positive endocardial cells (arrowheads) in the trabecular zone of *FOXO1*$^{EC\text{-}CA}$ embryos at E11.5 than those in the control. (G) Quantification of BrdU-positive nuclei as a percentage of total nuclei in myocardium indicated that *FOXO1*$^{EC\text{-}CA}$ displayed higher proliferation rates of CMs in the trabecular zone at E11.5 (n=5 per group). *p = 0.012, 2-way ANOVA. (H) Staining of *FOXO1*$^{EC\text{-}CA}$ heart sections at E11.5 for FOXO1 confirming that over-expressed FOXO1 demonstrated nuclear localization (arrowheads), while WT FOXO1 still displayed nucleocytoplasmic localization in a few EdCs (arrows). (I,J) Staining of *FOXO1*$^{EC\text{-}CA}$ heart sections at E10.5 with GFP (red) showing variations on the efficiency of FOXO1 over-expression in ventricular EdCs of different embryos. Arrows, representative GFP positive EdCs. DAPI was used to counter stain nucleus in all images. Scale bars: E,F,H, 50 µm; others, 100 µm.

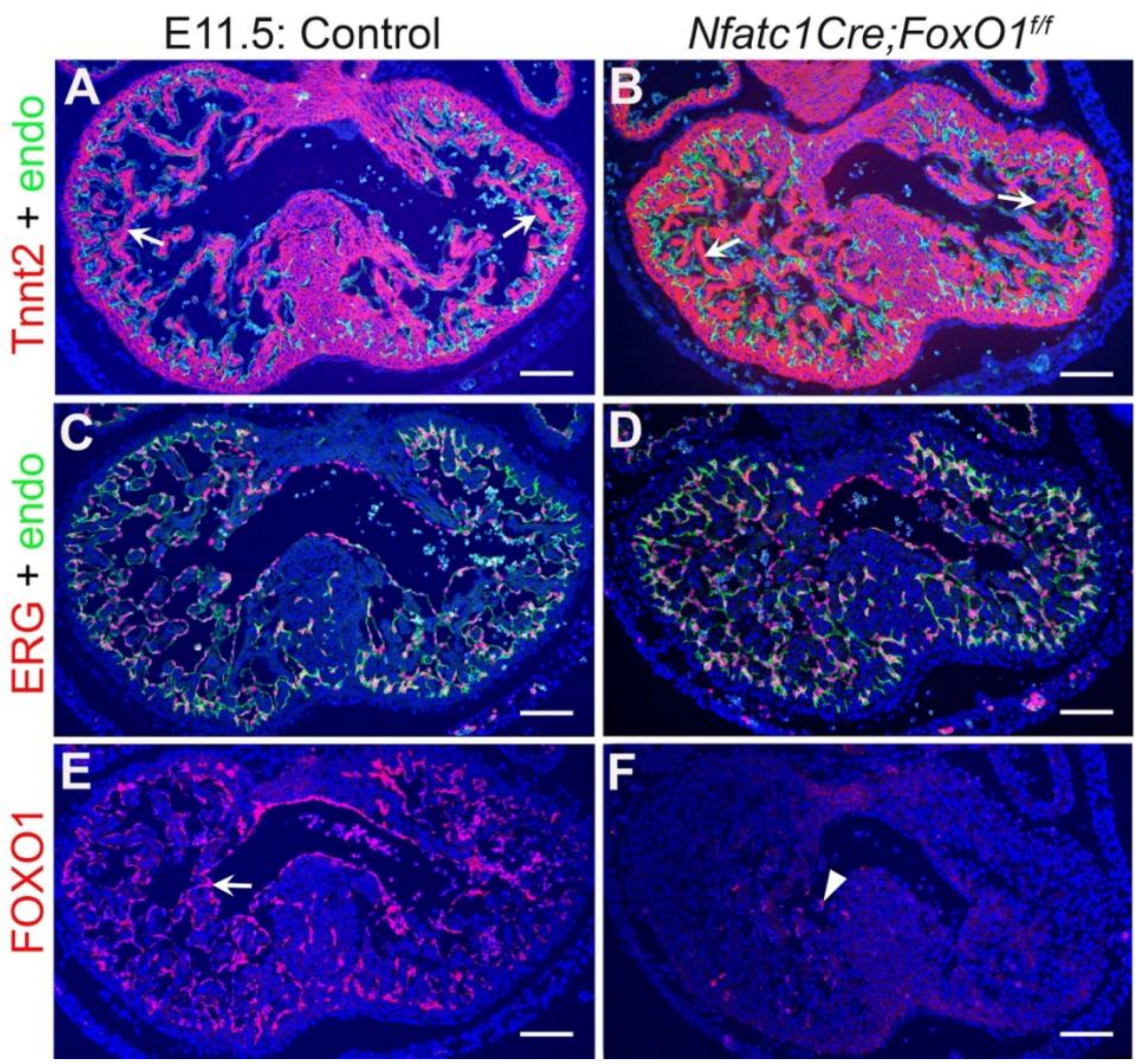

Fig. S6. Normal endocardial growth and trabeculation in endocardial *FoxO1* knockout mice. (A-D) Control (*FoxO1*$^{f/f}$ and *FoxO1*$^{f/+}$) and *Nfatc1*^{Cre}*;FoxO1*$^{f/f}$ consecutive ventricular sections at E11.5 were co-stained for troponin T (Tnnt2)/endomucin (endo) or ERG/endomucin, showing normal trabeculae (arrows) and endocardial network (visualized by staining with endomucin) with normal number of EdCs (ERG+ nuclei) in both control and mutant hearts. (E,F) Immunostaining for FOXO1 (red) showing only minimal and sporadic FOXO1 staining (arrowhead) in the mutant endocardium, in contrast to robust staining in the control (arrow). DAPI was used to counter stain nucleus in all images. Scale bars: 100 µm.

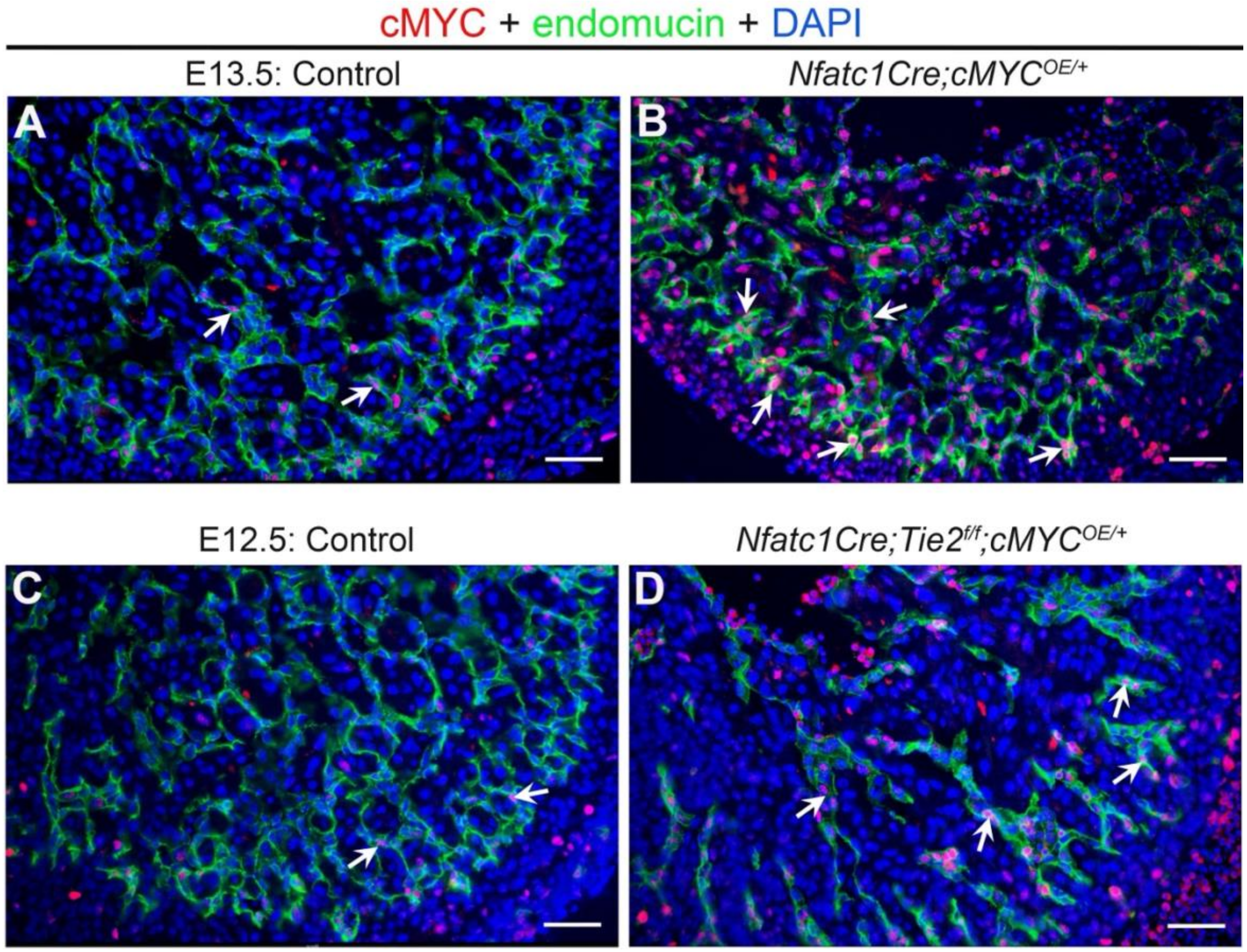

Fig. S7. Cardiac defects in *Tie2-cko* mice were not rescued by endocardial forced activation of cMYC. (A,B) Co-immunostaining of control (*cMYC*$^{OE/+}$) and *Nfatc1*^{Cre}*;cMYC*$^{OE/+}$ ventricular sections for cMYC and endomucin, showing much higher cMYC staining (arrows) in mutant EdCs than that in control EdCs, indicating successful and efficient *Nfatc1*^{Cre} mediated cMYC overexpression in the mutant endocardium, although the endocardial network in both control and mutant hearts at E13.5 were normal. (C,D) Co-immunostaining of control and *Nfatc1*^{Cre}*;Tie2*$^{f/f}$*;cMYC*$^{OE/+}$ heart sections at E12.5 for cMYC and endomucin, showing simplified endocardial network (visualized by staining with endomucin) in the double mutant ventricles compared to the control (*Tie2*$^{f/f}$ and *Tie2*$^{f/f}$*;cMYC*$^{OE/+}$). DAPI was used to counter stain nucleus in all images. Scale bars: 50 µm.

Development • Supplementary information

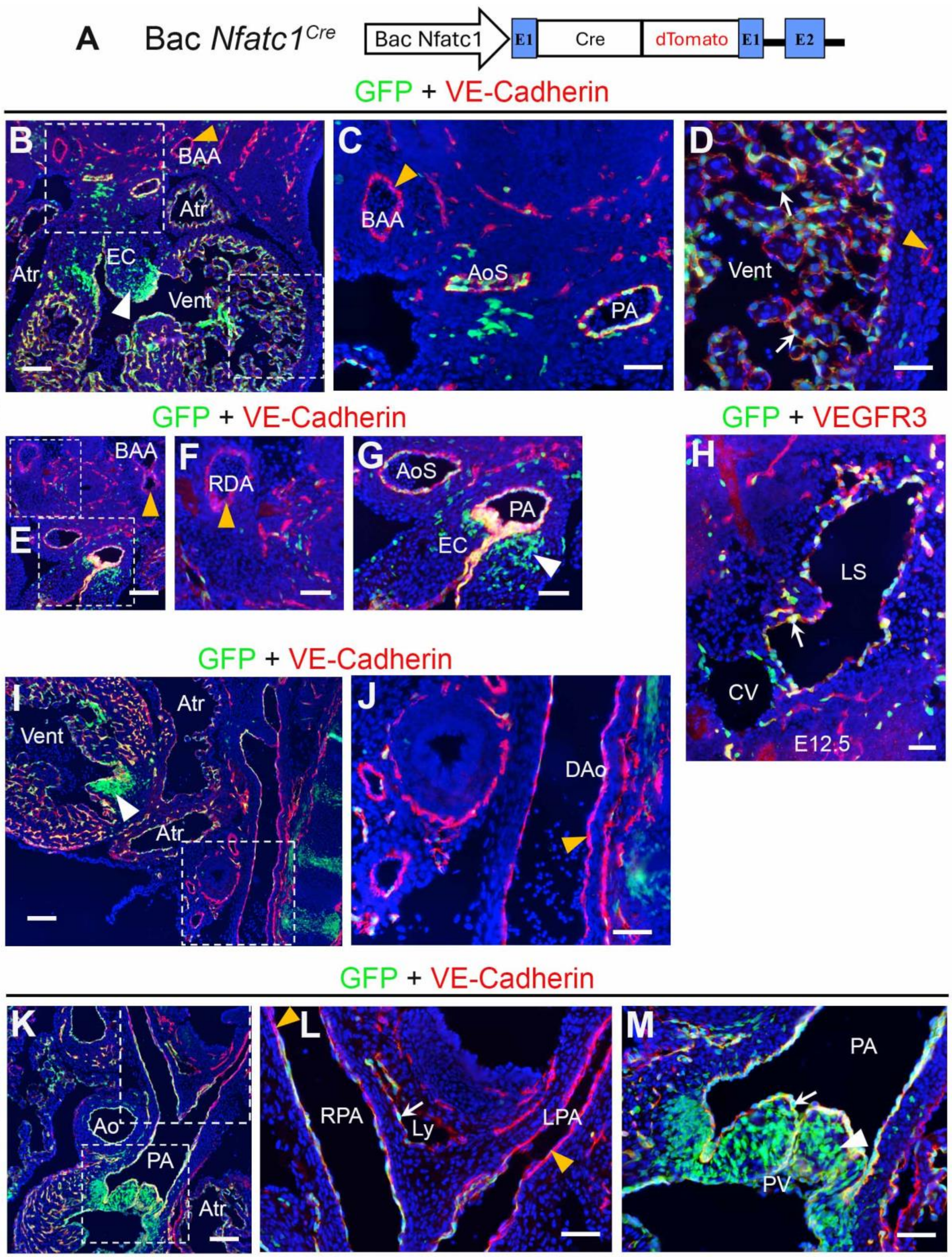

Development • Supplementary information

Fig. S8. Generation and characterization of a Bac *Nfatc1*^{Cre} transgenic mouse line. (A) Schematic overview of Bac *Nfatc1*^{Cre} transgenic mouse line generated via BAC recombineering. (B-M) Lineage tracing of *Nfatc1*^{CreBac}*;RCE*^{fsEGFP} embryos at indicated stages. (C, D), (F, G), (J) and (L, M) are the higher magnification images of the white squared areas in (B), (E), (I) and (K), respectively. *Nfatc1*^{CreBac} drives robust recombination specifically throughout the atrial and ventricular endocardium in addition to cushion endocardium (arrows), endocardial EMT-derived mesenchymal cells (arrowheads) and VEGFR3+ lymphatic ECs (arrow), but it is not active in the endothelial cells that line the branchial arch arteries, dorsal aorta and peripheral vessels (yellow arrowheads) at E12.5. Analysis of *Nfatc1*^{CreBac}*;RCE*^{fsEGFP} embryos at E14.5 and at E16.5 reveals endothelial GFP expression in the proximal aorta and pulmonary artery but gradually demolishing in the descending aorta and more distal pulmonary arteries. Atr, atrium; Ao, aorta; AoS, Aortic Sac; BAA, branchial arch artery; CV, cardinal vein; DAo, descending aorta; EC, endocardial cushion; LPA, left pulmonary artery; LS, lymphatic sac; Ly, lymphatic vessels; PA, pulmonary artery; PV, pulmonary valve; RDA, right dorsal aorta; RPA, right pulmonary artery; Vent, ventricle. DAPI was used to counter stain nucleus in all images. Scale bars: B, E, I and K, 100 µm; others, 50 µm.

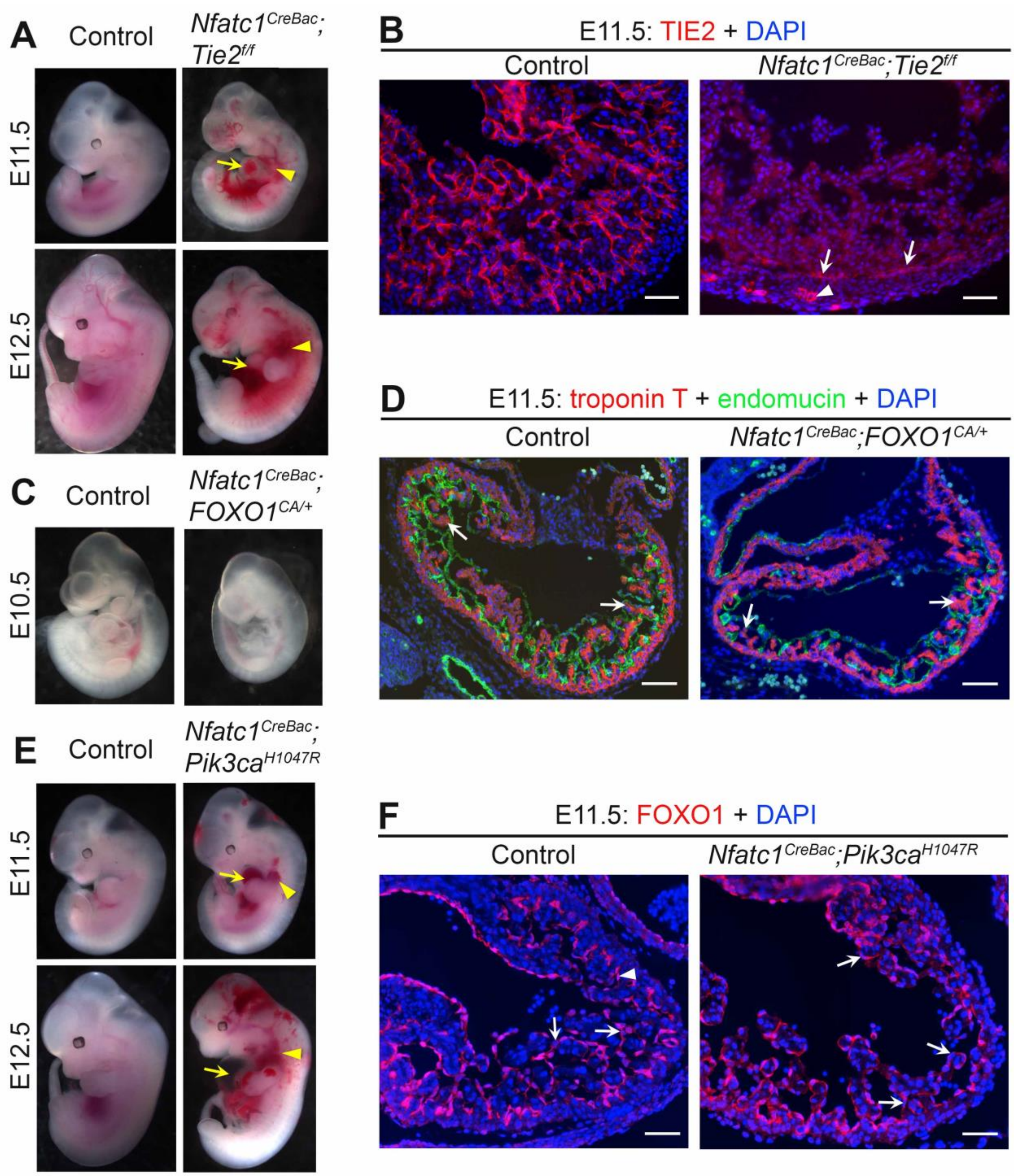

Development • Supplementary information

Fig. S9. The Bac *Nfatc1^{Cre}* transgenic mouse line is more efficient than the knock-in *Nfatc1^{Cre}* line. (A) Gross images of embryos showing that *Nfatc1^{CreBac};Tie2$^{f/f}$* embryos developed pericardial effusions (arrows) and perivascular hemorrhage (arrowheads) at E11.5 and complete lethality at E12.5, compared with normal control (*Tie2$^{f/+}$* and *Tie2$^{f/f}$*). (B) Left ventricular sections of control and *Nfatc1^{CreBac};Tie2$^{f/f}$* embryos at E11.5 were stained with anti-TIE2 antibody, showing specific and almost complete TIE2 deletion in EdCs without change of normal expression of TIE2 in epicardial (arrows) or blood vessel ECs (arrowhead). (C) Compared with littermate control (*FOXO1$^{CA/+}$*), *Nfatc1^{CreBac}*;*FOXO1$^{CA/+}$* embryos at E10.5 were runted. (D) Control and *Nfatc1^{CreBac}*;*FOXO1$^{CA/+}$* ventricular sections at E10.5 were stained for troponin T and endomucin, showing abnormal trabeculae (arrows) and endocardial network in the mutant heart. (E) *Nfatc1^{CreBac}*;*Pik3ca^{H1047R}* embryos developed pericardial effusions (arrows) and perivascular hemorrhage (arrowheads) at E11.5 and complete lethality at E12.5. (F) Control (*Pik3ca$^{H1047R/+}$*) and *Nfatc1^{CreBac}*;*Pik3ca^{H1047R}* ventricular sections at E11.5 were stained with FOXO1 showing that compared with clear nuclear localization of FOXO1 (arrowhead) in a small percentage of EdCs of the control, almost all EdCs of the mutant displayed a diffuse nucleocytoplasmic localization of FOXO1 (arrows). DAPI was used to counter stain nucleus in all images. Scale bars: D, 100 μm; others, 50 μm.

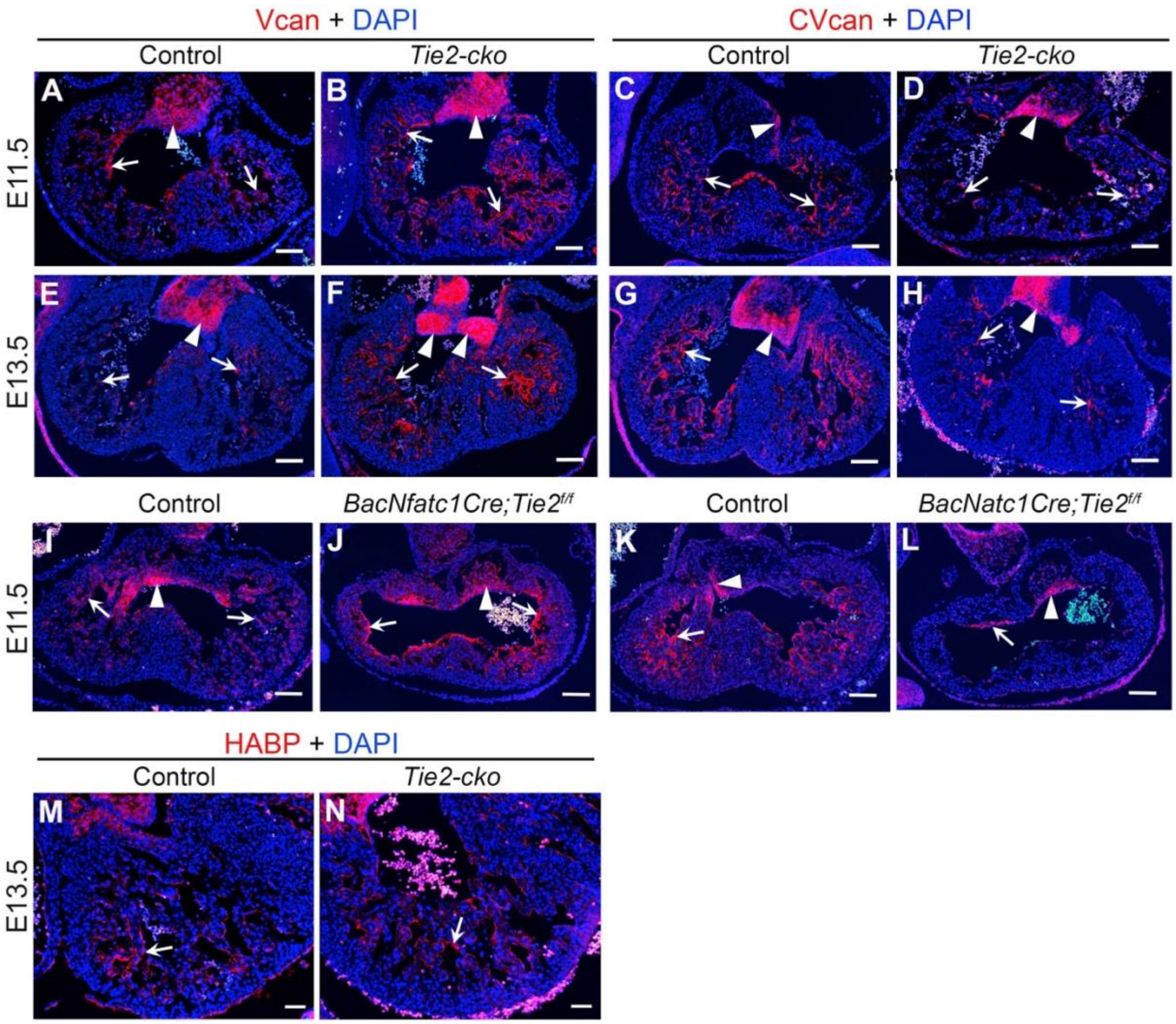

Fig. S10. Endocardial loss of *Tie2* led to abnormal cardiac ECM composition and metabolism. (A-C) Control and *Tie2* mutant heart sections at indicated stages were stained for intact versican (Vcan), cleaved Vcan (CVcan), and HABP (M,N) showing that compared to their littermate controls, the expression of Vcan was significantly increased in *Tie2-cko* and *BacNfatc1Cre*;*Tie2*$^{f/f}$ ventricles, whereas CVcan was dramatically reduced, but no significant alteration on the expression of HABP (staining hyaluronan). Arrows, positive staining in ventricles; arrowheads, positive staining in AVC cushions. DAPI was used to counter stain nucleus in all images. Scale bars: A-L, 100 μm; others, 50 μm.

Development • Supplementary information

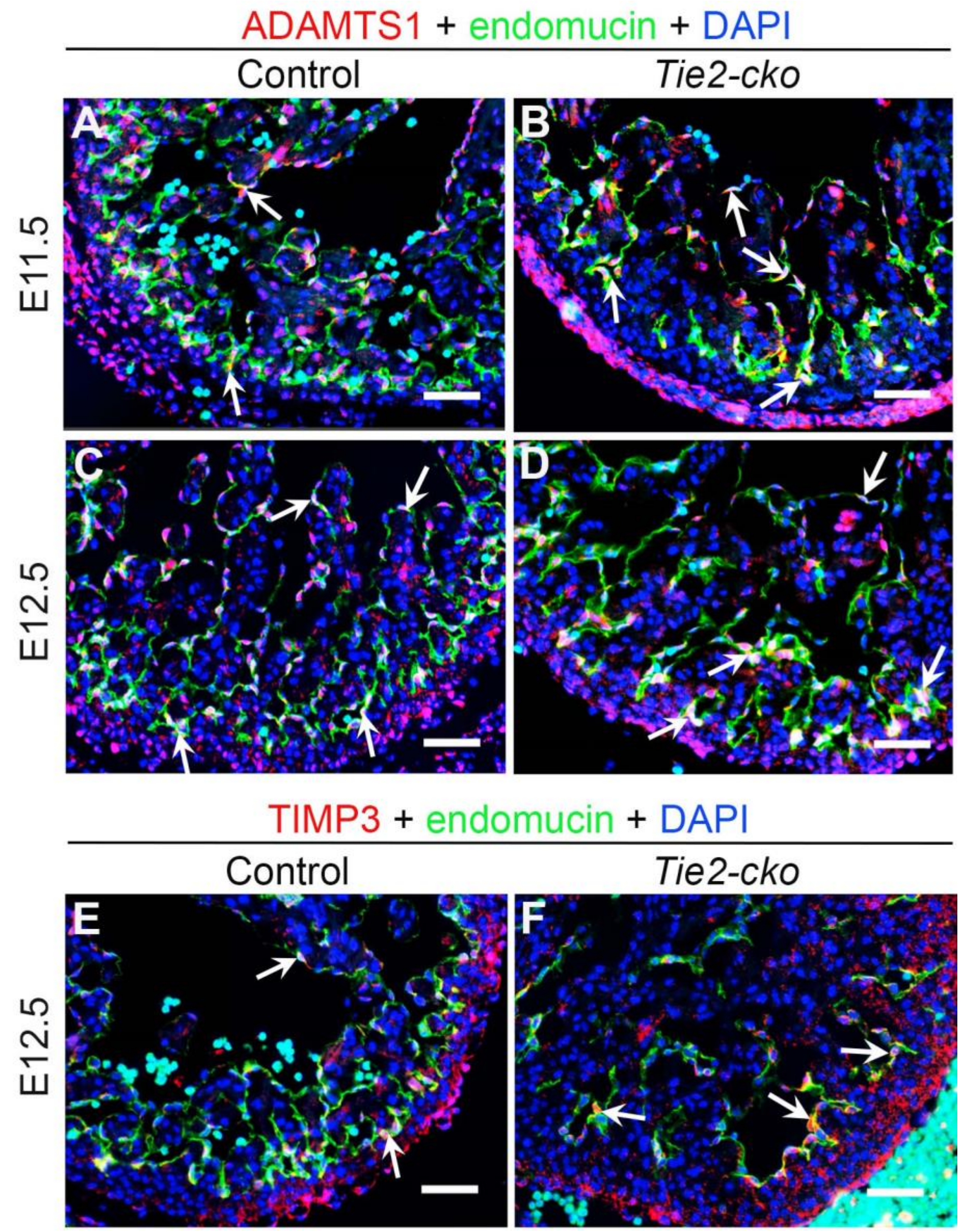

Fig. S11. Endocardial loss of *Tie2* led to enhanced expression of ADAMTS1 and TIMP3 in *Tie2-cko* ventricles. (A-F) Control and *Tie2-cko* ventricular sections at E11.5 and E12.5 were co-stained for endomucin (green) and ADAMTS1 (red, A-D) or TIMP3 (red, E-F). Arrows, representative positive staining in ventricular EdCs. DAPI was used to counter stain nucleus in all images. Scale bars: 50 μm.

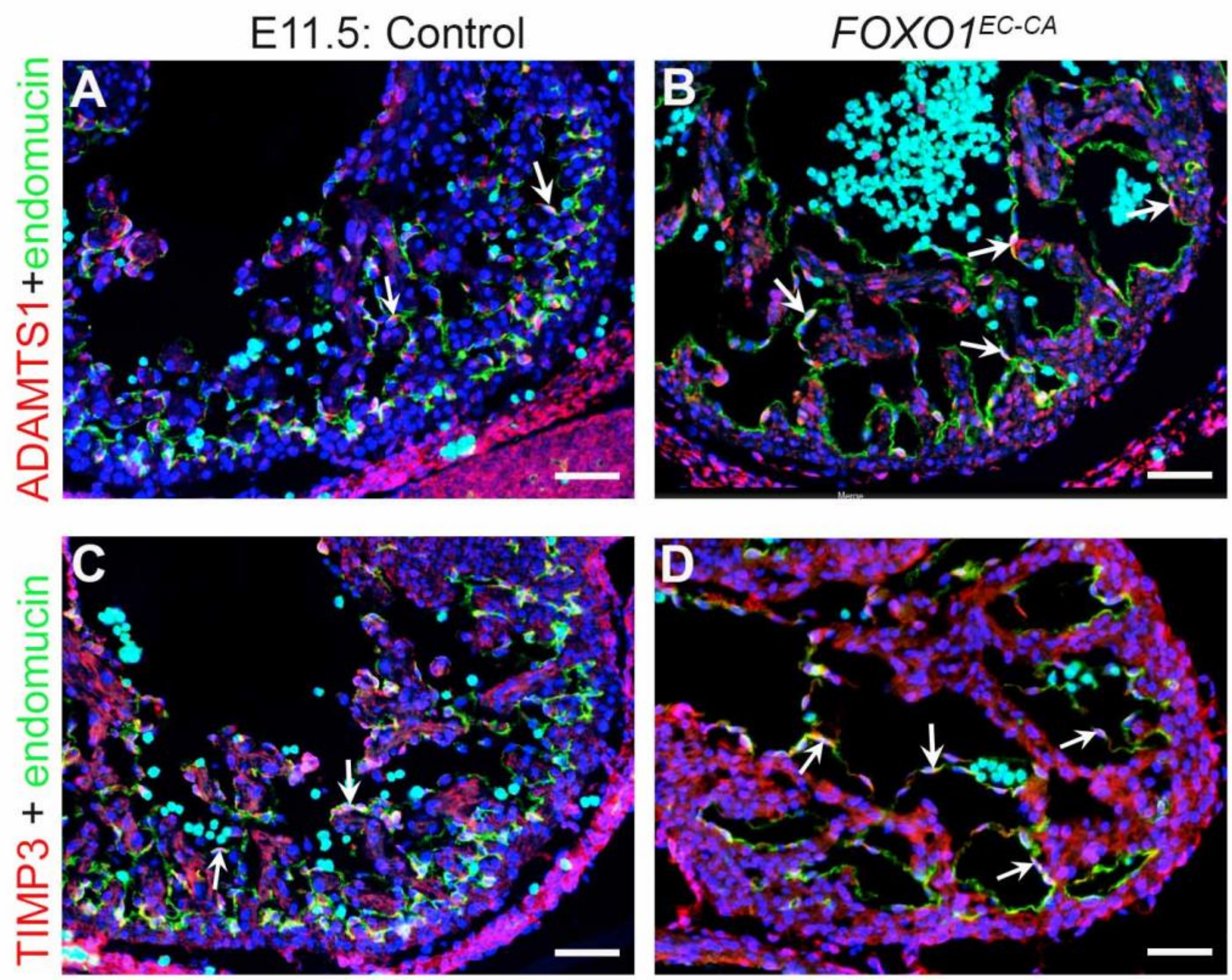

Fig. S12. Endocardial forced activation of FOXO1 led to enhanced expression of ADAMTS1 and TIMP3 during trabeculation. (A-D) Control and *Tie2-cko* ventricular sections at E11.5 were co-stained for endomucin (green) and ADAMTS1 (red, A,B) or TIMP3 (red, C,D). Arrows, representative positive staining in ventricular EdCs. DAPI was used to counter stain nucleus in all images. Scale bars: 50 μm.

Development • Supplementary information

Table S1. Sources of primary antibodies

Antibody	Supplier	Catalogue #	Application
Hyaluronic acid binding protein, biotinylated	Millipore	385911	IHC 1:200
Goat anti-mouse Tie2	R&D Systems	AF762	IHC 1:200
Goat anti-Flk1 (VEGFR2)	R&D Systems	AF644	IHC 1:200
Goat anti-VE-Cadherin	R&D Systems		
Rhodamine conjugated human anti-tubulin (Clone AbD22584)	Bio-Rad	12004166	WB 1:1000
Mouse anti-Tnnt2 Antibody	Developmental Studies Hybridoma Bank	CT3	IHC 1:2
Rabbit anti-Ki67	abcam	ab15580	IHC 1:500
Rabbit anti-Phospho-YAP (D9W2I)	Cell Signaling	#13008	IHC 1:10,000*
Rabbit anti-YAP (D8H1X)	Cell Signaling	#14074	IHC 1:2,000*
Rabbit anti-Cyclin D1 (SP4)	ThermoFisher	MA5-14512	IHC 1:5,000*
Rabbit anti-Phospho-Akt (Ser473)	Cell Signaling	#4060	IHC 1:800*
Rabbit anti-FOXO1	Cell Signaling	2880	1:200
Rabbit anti-ERG1	abcam	ab92513	IHC 1:100
Rabbit anti-Versican	Chemicon	AB1033	IHC 1:1500
Rabbit anti-Versican V0,V1 Neo (DPEAAE)	ThermoFisher	PA1-1748A	IHC 1:1500<br>WB 1:2000
Rabbit anti-MYC	Millipore	#06-340	IHC 1:1000*
Rabbit anti-TIMP3	ThermoFisher	PA5-116048	IHC 1:100*
Rat anti-endomucin	abcam	ab106100	IHC 1:200
Rat anti-BrdU	abcam	ab6326	IHC 1:100
Rabbit anti-GFP	Invitrogen	A11122	IHC 1:200
Sheep anti-ADAMTS1	ThermoFisher	PA5-47790	IHC 1:300*

WB: Western blot; IHC: Immunohistochemistry; *TSA

Development • Supplementary information

Table S2. Sequences of qPCR primers

Gene	Forward primer	Reverse primer
18S	AGGAATTCCCAGTAAGTGCG	GCCTCACTAAACCATCCAA
Vcam1	AAGAGAACCCAGGTGGAGGT	GGGGCAACGTTGACATAAAG
Pdgfb	AAGTGTGAGACAATAGTGACCCC	CATGGGTGTGCTTAAACTTTCG
Angpt2	GGTTGCTATCCGTAAAGAAGAGC	GGGGAAGGTCAGTGTGTAGATG
Esm1	TGCTACCGTACAGTCTCAGG	ACCAAACTCGTCACCAAAATCAT
Nos3	TGAAGATCTCTGCCTCACTCATG	AGTCTCAGAGCCATACAGGGTT
Cx37(Gja4)	TCCCACATCCGATACTGGGT	CCCGCCGAGACAGGTAGAT
Col13a1	GGAGCACCTGGACTAGACG	GCCTTGGACTGGTAAGCCAT
Timp3 (P0)	AGCTGTTCCTCTACCCCTGT	GCCTCGTGGATTTACAGGCT
Timp3 (P1)	GAGGAGCAGCTGACAACCTT	TCTGATGCTGAAGGAACACG
Timp3 (P2)	GCCAGTGCATACATACAGGTC	TGTCTCGAAAAACCGAAAAA
Timp3 (P3)	GATTGAGTTGTGGGGCAAAG	CCTGGCAAAACAAACTGCTT

Development • Supplementary information

Table S3. Survival of genetically modified embryos

Nfatc1^{CreBac};Tie2$^{f/+}$ X *Tie2$^{f/f}$*

Genotype	E10.5	E11.5	E12.5	E13.5
Tie2$^{+/f}$	11	10	13	13
Tie2$^{f/f}$	6	16	10	10
Nfatc1^{CreBac};Tie2$^{f/+}$	7	15	9	8
Nfatc1^{CreBac};Tie2$^{f/f}$	8 (0)	13 (1)	11 (11)	7 (7)
Total	32	54	43	38

Nfatc1^{Cre} X *FOXO1$^{CA/CA}$*

Genotype	E9.5	E10.5	E11.5	E12.5	E13.5	E14.5-E15.5	E16.5-E18.5
FOXO1$^{CA/+}$	11	18	24	29	41	45	51
Nfatc1^{Cre};FOXO1$^{CA/+}$ (*FOXO1^{EC-CA}*)	14 (0)	22 (0)	23 (1)	25 (3)	38 (12)	40 (23)	46 (32)
Total	25	40	47	54	79	85	97

Nfatc1^{CreBac} X *FOXO1$^{CA/CA}$*

Genotype	E10.5	E11.5	E12.5	E13.5	E14.5	E16.5
FOXO1$^{CA/+}$	8	16	20	17	15	24
Nfatc1^{CreBac};FOXO1$^{CA/+}$	10*	19 (18)	18 (16)	14 (13)	12 (11)	22 (22)
Total	18	35	38	31	27	46

*50% (5/10) were runted embryos.

Nfatc1^{Cre} X *Pik3ca$^{H1047R/H1047R}$*

Genotype	E11.5	E12.5	E13.5	E14.5	E15.5
Pik3ca$^{H1047R/+}$	15	23	23	30	15
Nfatc1^{Cre};Pik3ca$^{H1047R/+}$	15 (0)	19 (3)	38 (11)	27 (17)	12 (12)
Total	35	42	49	57	27

Nfatc1^{CreBac} X *Pik3ca$^{H1047R/H1047R}$*

Genotype	E10.5	E11.5	E12.5	E13.5
Pik3ca$^{H1047R/+}$	8	15	14	27
Nfatc1^{CreBac};Pik3ca$^{H1047R/+}$	9 (0)	13 (0)	18 (18)	12 (12)
Total	17	28	32	39

** All mutants are showing as total number (dead number).

Development • Supplementary information

Table S4. Expression of selected genes in E10.5 wild-type and *Tie2-cko* ventricular EdCs

Genes	Tie2cko_avg	Ctrl_avg	avg_log2FC	p_val	p_val_adj
Tek (Tie2)	0.16335205	0.97269103	0.76187727	1.14E-48	2.21E-44
Gja4	1.18278617	2.25264398	0.57544211	2.63E-19	5.10E-15
Cdkn1c	29.6629432	40.3080536	0.4299269	1.61E-04	1.00E-02
Nppa	2.19954926	3.02204764	0.33006149	7.62E-05	1.04E-02
Edn1	0.82026974	0.22525161	-0.5710742	9.42E-18	1.83E-13
Ptn	3.12838575	1.28879217	-0.8509913	1.35E-06	2.62E-03
Tcf4	1.30994059	0.76381156	-0.3891593	3.66E-08	7.10E-04
Egr1	0.70821271	0.42515793	-0.2613658	5.52E-03	1
Pdgfb	0.1521337	0.0409437	-0.1464162	4.53E-06	8.78E-02
Emcn	8.10090899	5.17914081	-0.5586044	1.37E-13	2.66E-09
Plvap	4.032612	4.994605	0.2523573	2.71E-07	5.25E-03
Col13a1	0.3657591	0.802746	0.40049311	3.21E-14	6.22E-10
Col3a1	1.99517971	2.63020196	0.27740725	5.03E-06	9.75E-03
Col26a1	0.35234051	0.68515659	0.31742419	2.04E-09	3.96E-05
Hbegf	0.43749468	0.14036305	-0.3340634	3.60E-10	6.98E-06
Tgfbi	0.56247061	0.21966184	-0.3573478	8.58E-10	1.66E-05
Ccdc80	1.6965806	0.98587037	-0.4413597	3.05E-09	5.91E-05
Itga1	1.139647	0.6680866	-0.3591786	1.32E-07	2.55E-03
Sparc	7.47608778	5.90178876	-0.2964282	1.44E-07	2.79E-03
S100a6	0.55036296	0.22787937	-0.3364372	1.20e-05	2.32E-01
Vcan	0.6162167	0.3942759	-0.2131046	5.05e-03	1
Hapln1	3.00654177	4.36994637	0.42255016	1.20E-13	2.33E-09
Adamts1	0.65915738	0.39353515	-0.2517013	7.62E-05	1
Adamts4	0.00262831	0.00397035	0.00192978	5.96E-01	1
Adamts5	0.03404816	0.05161858	0.02430815	8.02E-01	1
Adamts9	0.1206122	0.1293753	0.01123791	8.86E-02	1.72E-01
Adamts10	0.2635967	0.2489138	-0.0168621	1.14E-01	1
Timp1	0.4037647	0.4175457	0.01409416	7.42E-02	1
Timp2	0.4963451	0.400412	-0.0955916	6.34E-02	1
Timp3	0.424627	0.2043927	-0.2422784	2.88E-02	1
Sox7	0.2426721	0.1821502	-0.0720324	4.78E-01	1
Arap3	0.4271005	0.4449746	0.01795719	1.61E-02	1
Tmem100	0.633603	0.8364115	0.168832	1.24E-01	1
Npr3	1.84443	2.357696	0.2393317	6.10E-01	1
Nfatc1	0.4437704	0.3886983	-0.0561082	5.43E-02	1
Gpr126(Adgrg6)	1.082964	1.300996	0.1436208	8.83E-01	1
Rasip1	1.103418	1.161516	0.03930764	5.61E-02	1
Kdr	1.9849506	1.76509134	-0.1103799	1.28E-01	1
Vwf	0.2711311	0.4432142	0.1831726	8.93E-02	1
Cdh5	2.230009	2.327156	0.04275134	6.08E-02	1
Gata2	0.2050618	0.2253252	0.02405753	2.79E-01	1

Genes in red, upregulated in *Tie2-cko* EdCs; Genes in blue, downregulated in *Tie2-cko* EdCs.

Development • Supplementary information
