# Supplementary material for: Endocardial TIE2 orchestrates cardiac trabeculation via sequestering FOXO1 within the cytoplasm and stimulating versican cleavage
Source: Development. 2026 Jul 29;153(14):dev205674. doi: 10.1242/dev.205674 (PMC13450898; doi:10.1242/dev.205674)
Supplement: Supplementary file 2 [file develop-153-205674_review_history.pdf]

Endocardial Tie2 orchestrates cardiac trabeculation via sequestering FOXO1 within the cytoplasm and stimulating versican cleavage

H. Scott Baldwin, Kevin Tompkins, Yan Gong, Bryan Helm, Qi Liu and Xianghu Qu
DOI: 10.1242/dev.205674

Editor: Benoit G. Bruneau

Review timeline

Original submission:	17 March 2026
Editorial decision:	24 April 2026
First revision received:	9 June 2026
Accepted:	11 June 2026

Original submission

First decision letter

MS ID#: dev.205674

MS TITLE: Endocardial Tie2 orchestrates cardiac trabeculation via sequestering FOXO1 within the cytoplasm and stimulating versican cleavage

AUTHORS: Xianghu Qu, H. Scott Baldwin, Kevin Tompkins, Yan Gong, Bryan Helm and Qi Liu

Dear Dr Qu,

I have now received all the referees reports on the above manuscript, and have reached a decision. The referees' comments are appended below.

The overall evaluation is positive and we would like to publish a revised manuscript in Development, provided that the referees' comments can be satisfactorily addressed. Please attend to all of the reviewers' comments in your revised manuscript and detail them in your point-by-point response. If you do not agree with any of their criticisms or suggestions explain clearly why this is so. If it would be helpful, you are welcome to contact us to discuss your revision in greater detail. Please send us a point-by-point response indicating your plans for addressing the referees' comments, and we will look over this and provide further guidance.

Reviewer 1

SUMMARY OF THE ADVANCE MADE IN THIS PAPER AND ITS POTENTIAL SIGNIFICANCE TO THE FIELD

Baldwin et al present an important, embryonic mouse genetic study that interrogates the downstream mechanism of endocardial TIE2 signaling. They confirm a longstanding hypothesis linking TIE2 to FOXO1 inactivation/nuclear exclusion. Genetic loss of endocardial TIE2 increases nuclear FOXO1 and reduces endocardial cell proliferation, while constitutively active FOXO1 phenocopies and loss of FOXO1 partially rescues the endocardial TIE2 KO phenotype. To understand the mechanism downstream of FOXO1, they performed single cell sequencing and find that versican metabolism is altered, potentially explaining the TIE2 KO hyper trabeculation phenotype.
These studies are well performed with appropriate controls and presented nicely.

© 2026. Published by The Company of Biologists under the terms of the Creative Commons Attribution License (https://creativecommons.org/licenses/by/4.0/).

SUGGESTIONS TO AUTHORS

Some questions raised by their findings are outlined below.
1. Is TIE2 regulation of FOXO1 conserved in other endothelial cells? Addressing this question would substantially add to the significance of the work as the links between TIE2 and FOXO1 have been circumstantial up to this point through in vitro studies and interrogation of upstream ligands.

2. Does loss of FOXO1 and gain of FOXO1 alter ADAMTS1 TIMP3 expression? This link to versican proteolysis is interesting with potential clinical relevance, should be interrogated, and could be readily performed with existing samples.
a. What is the connection of FOXO1-VCAN signaling to retinoic acid signaling? The authors previously reported the endocardial TIE2 KO phenotype being dependent on retinoic acid signaling in the myocardium. Is VCAN proteolysis upstream of these myocardial phenotypes?

3. Is it TIE2 activation or inactivation that drives FOXO1 nuclear exclusion? While definitively addressing this question is out of the scope of this manuscript, others have used phospho-TIE2 staining as a surrogate marker for RTK activation (Kraft…Makinen NCVR 2025 and Li…Kahn JEM 2026).
Other points:
1. More detailed/careful lineage tracing of the Nfatc1-BAC-Cre and explanation of phenotypic severity. The authors discuss an important limitation regarding the Nfatc1-ires-Cre. Detailed lineage tracing of the BAC-Cre is needed to ensure endocardial-lineage fidelity and not "leak" in other cells/tissues for example the aorta, pharyngeal arch vasculature. Is Cre expression higher in the BAC? Is the reason for a more severe phenotype more complete TIE2 expression loss? In the same line of thought, could a weak Nfatc1-ires-Cre be the reason why an early endocardial VEGFR2 KO phenotype is not appreciated? Additional lineage tracing data and characterization of this new Cre is the most important new data required for the manuscript.

2. This manuscript focuses on myocardial trabeculae and the overlying endocardium. Are there any defects in the valve/cushion endocardium that are also rich in versican? Conserved TIE2-FOXO1 signaling?

Reviewer 2

SUMMARY OF THE ADVANCE MADE IN THIS PAPER AND ITS POTENTIAL SIGNIFICANCE TO THE FIELD

In the manuscript by Baldwin et al., the authors build on their previous finding that endocardial Tie2 is required for cardiac trabeculation by examining the underlying mechanism. The authors show that the nuclear localisation of FoxO1 is dramatically increased in Tie2 cKO endocardial cells and that this is associated with a decrease in pAkt. Overexpression of a CA-FoxO1 variant replicates the Tie2 cKO phenotype, and FoxO1/Tie2 double cKO display a partial rescue. However, most double cKO embryos die at the same time as Tie2 cKO embryos and hyperactivation of pAkt does not rescue the trabeculation phenotype in Tie2 cKO embryos. The authors observe a decrease in Cyclin D1, cMYC, and pYAP in Tie2 cKO endocardial cells, suggesting that endocardial proliferation is inhibited. However, overexpressing cMyc in endocardial cells does not rescue the trabeculation defect in Tie2 cKO embryos. scRNA-seq identified an increase in Vcan expression in Tie2 cKO embyros, which is validated with a Vcan antibody. However, cleaved Vcan staining is decreased. The authors also observed upregulation of Adamts1 and Timp3 in the scRNA-seq data, and identify FoxO1 binding sites in Timp3 that they validate by ChIP-seq in cardiac endothelial cells. They propose a model whereby FoxO1 activity is regulated by Tie2 signalling through the PI3K/Akt pathway. FoxO1 then directly regulates Timp3 expression to regulate Vcan cleavage, thereby affecting cardiomyocyte proliferation and leading to the observed trabeculation defect.

The findings presented by the authors are novel and interesting, and the proposed mechanism significantly advances our understanding of the mechanism by which Tie2 signalling leads to trabeculation defects. However, there are some holes in the proposed mechanism that should be addressed to strengthen this claim.

SUGGESTIONS TO AUTHORS

© 2026. Published by The Company of Biologists under the terms of the Creative Commons Attribution License (https://creativecommons.org/licenses/by/4.0/).

Major:
1. Is each data point in the various quantification plots from an independent embryo? The quantification section in the methods mentions "six embryos of each genotype" were analysed, but all plots only have five data points. Please clarify.

2. Line 187 refers to fig 4, but the data is in fig 3. Please correct. It is also not clear what control was used in this experiment, please clarify their genotype.

Presumably the double cKO embryos examined in fig 3 (at E14.5) have a mild phenotype as the authors say that the majority die by E13.5 (line 182). Did the authors examine the phenotype in E13.5 double cKO embryos? Is the Tie2 phenotype also milder in those younger double cKOs? The E13.0 double cKO in fig S4B looks more severe than the E14.5 embryo shown in fig 3F. It would be good to include more data at the earlier stages to get a better sense of how much of the Tie2 phenotype one can attribute to FoxO1 activation and how much is due to other effectors of Tie2 signalling.

3. I'm confused how the trabeculation defect observed in the PI3K hyperactivation embryos in fig 5 fits with the rest of the manuscript. Presumably the lethality/trabeculation defect in these embryos is not due to the proposed FoxO1/Timp3/Vcan pathway as one would expect FoxO1 to be phosphorylated and localised to the cytoplasm in these embryos (as in WT controls). Did the authors examine FOXO1 phosphorylation and/or localisation in the PI3K hyperactivation embryos?

4. Line 473 in the discussion mentions the use of the ChIP-qPCR experiments to "validate that TIMP3 is a direct target of FOXO1 in developing endocardium". However, an important caveat here is that these experiments were performed using a cardiac endothelial cell line rather than endocardial cells. While I understand there are technical challenges to performing this experiment with endocardial cells, the fact that the validation of Timp3 and as a direct target of FoxO1 was not performed in endocardial cells should be made clear in the discussion.

5. The model in fig 9 proposes that cardiomyocyte proliferation is downstream of the proposed Tie2/FoxO1/Timp3/Vcan pathway. However, cardiomyocyte proliferation was not examined in any of the experiments in this manuscript. I assume this point is a reference to the increased proliferation the authors have previously reported in Tie2 cKOs. Have the authors examined cardiomyocyte proliferation/cell number in the CA-FOXO1 or double cKO embryos? Direct evidence linking the proposed pathway to cardiomyocyte proliferation is lacking and this should be made clear in the model. The authors may already have the samples to perform such an analysis that would test this claim.

Minor:
1. Please consider reporting P-values in full to allow for more accurate interpretation of results (see https://www.nature.com/articles/d41586-019-00857-9).

2. I understand the desire to present as much data as possible, and the authors have generated a lot of it, but nine figures is extensive and the main findings are well represented in figs 1, 3, and 7. Would the authors consider moving some of the other figures to the supplement? E.g., fig 2 and 4, and maybe even 5 and 6.

3. It is unclear to me what the significance is of the E16.5 control data in figure 1E. Given that the Tie2-cKO embryos do not survive past E13.5.

4. It isn't clear what is meant by the sentence on line 136, "although no significant changes were detected on YAP expression". Please clarify this statement and what data this refers to.

5. In table S3, should the FOXO1 CA/CA genotype be CA/+? Line 155 refers to mutants, I assume this means heterozygotes rather than homozygotes? Similarly, the Pik3ca H1047R and Nfatc1 Cre embryos in table S3 would all be heterozygotes, no? Please clarify.

© 2026. Published by The Company of Biologists under the terms of the Creative Commons Attribution License (https://creativecommons.org/licenses/by/4.0/).

First revision

Author response to reviewers' comments

Reviewer 1: SUMMARY OF THE ADVANCE MADE IN THIS PAPER AND ITS POTENTIAL SIGNIFICANCE TO THE FIELD

Baldwin et al present an important, embryonic mouse genetic study that interrogates the downstream mechanism of endocardial TIE2 signaling. They confirm a longstanding hypothesis linking TIE2 to FOXO1 inactivation/nuclear exclusion. Genetic loss of endocardial TIE2 increases nuclear FOXO1 and reduces endocardial cell proliferation, while constitutively active FOXO1 phenocopies and loss of FOXO1 partially rescues the endocardial TIE2 KO phenotype. To understand the mechanism downstream of FOXO1, they performed single cell sequencing and find that versican metabolism is altered, potentially explaining the TIE2 KO hyper trabeculation phenotype.
These studies are well performed with appropriate controls and presented nicely.

Response: We thank the reviewer for the positive assessment of our manuscript and for recognizing the value this study.

SUGGESTIONS TO AUTHORS
Some questions raised by their findings are outlined below.
1. Is TIE2 regulation of FOXO1 conserved in other endothelial cells? Addressing this question would substantially add to the significance of the work as the links between TIE2 and FOXO1 have been circumstantial up to this point through in vitro studies and interrogation of upstream ligands.

Response: Yes, TIE2 regulation of FOXO1 is conserved in vascular endothelial cells. We agree that addressing this question is necessary, and we have discussed this in lines 101-103, 431-437 in our manuscript.

2. Does loss of FOXO1 and gain of FOXO1 alter ADAMTS1 TIMP3 expression? This link to versican proteolysis is interesting with potential clinical relevance, should be interrogated, and could be readily performed with existing samples.

Response: Yes, we have performed more immunostainings with existing samples and found similar alterations on ADAMTS1 and TIMP3 expression in *FOXO1*$^{EC\text{-}CA}$ mutants (added in supplemental figure 12), which is consistent to that FOXO1 gain of function phenocopied the cardiac defects seen in EdC *Tie2*-deficient hearts. However, loss of FOXO1 did not alter ADAMTS1 or TIMP3 expression, which is consistent with the observation that *Nfatc1*^{Cre};*FoxO1*$^{f/f}$ mutants had a normal lifespan with normal endocardial growth and trabeculation. Therefore, given the abundance of data presented and the length of the current manuscript, we do not think that the ADAMTS1 or TIMP3 expression data for loss of FOXO1 model is necessary to be included.

a. What is the connection of FOXO1-VCAN signaling to retinoic acid signaling? The authors previously reported the endocardial TIE2 KO phenotype being dependent on retinoic acid signaling in the myocardium. Is VCAN proteolysis upstream of these myocardial phenotypes?

Response: We do not have evidence to support how FOXO1-VCAN signaling is connected to retinoic acid signaling, although we believe that both are important to cardiomyocyte proliferation, and retinoic acid signaling and VCAN proteolysis might just function parallelly in our mutant models. We have therefore removed any reference to an experimentally validated link between FOXO1-VCAN signaling and retinoic acid signaling. Again, investigation of this signaling pathway is beyond the scope of these studies.

3. Is it TIE2 activation or inactivation that drives FOXO1 nuclear exclusion? While definitively addressing this question is out of the scope of this manuscript, others have used phospho-TIE2 staining as a surrogate marker for RTK activation (Kraft…Makinen NCVR 2025 and Li…Kahn JEM 2026).

© 2026. Published by The Company of Biologists under the terms of the Creative Commons Attribution License (https://creativecommons.org/licenses/by/4.0/).

Response: Yes, TIE2 activation drives FOXO1 nuclear exclusion and we have discussed this in our original manuscript (lines 101-111, 428-432). We agree that phospho-TIE2 staining is a very useful surrogate marker for RTK activation and appreciate these excellent studies. These references are now included.

Other points:
1. More detailed/careful lineage tracing of the Nfatc1-BAC-Cre and explanation of phenotypic severity. The authors discuss an important limitation regarding the Nfatc1-ires-Cre. Detailed lineage tracing of the BAC-Cre is needed to ensure endocardial-lineage fidelity and not "leak" in other cells/tissues for example the aorta, pharyngeal arch vasculature. Is Cre expression higher in the BAC? Is the reason for a more severe phenotype more complete TIE2 expression loss? In the same line of thought, could a weak Nfatc1-ires-Cre be the reason why an early endocardial VEGFR2 KO phenotype is not appreciated? Additional lineage tracing data and characterization of this new Cre is the most important new data required for the manuscript.

Response: We thank the reviewer for these helpful comments and suggestions. Yes, we have provided additional lineage tracing data and characterization of this new Cre (new Fig.S8). Usually, a transgenic Bac-Cre line (which may carry multiple copies) is more efficient than a knock-in line (only one copy). It's difficult for us to provide evidence that Cre expression is higher in the BAC line but we show almost complete loss of TIE2 expression (Fig.S9B) which is in line with a more severe phenotype. During submission of this manuscript, we had used the *Nfatc1-BAC-Cre* to delete *Vegfr2* to obtain a few $Nfatc1^{CreBac}$;$Vegfr2^{f/f}$ embryos from 2 litters, but found no trabeculation or endocardial growth defects in mutant hearts (data not shown). In addition, our endocardial-specific deletion of *Vegfr2* via $Nfatc1^{Cre}$ did not lead to FOXO1 nuclear localization in EdCs. Therefore, the observation that an early endocardial VEGFR2 KO phenotype is not appreciated is probably not because Nfatc1-ires-Cre is weaker than the Bac line.

2. This manuscript focuses on myocardial trabeculae and the overlying endocardium. Are there any defects in the valve/cushion endocardium that are also rich in versican? Conserved TIE2-FOXO1 signaling?

Response: Yes, there are defects in cardiac valves that are also rich in versican from E14.5 and TIE2-FOXO1 signaling is conserved during cardiac valve remodeling. This is an ongoing project in our lab.

Reviewer 2: SUMMARY OF THE ADVANCE MADE IN THIS PAPER AND ITS POTENTIAL SIGNIFICANCE TO THE FIELD

In the manuscript by Baldwin et al., the authors build on their previous finding that endocardial Tie2 is required for cardiac trabeculation by examining the underlying mechanism. The authors show that the nuclear localisation of FoxO1 is dramatically increased in Tie2 cKO endocardial cells and that this is associated with a decrease in pAkt. Overexpression of a CA-FoxO1 variant replicates the Tie2 cKO phenotype, and FoxO1/Tie2 double cKO display a partial rescue. However, most double cKO embryos die at the same time as Tie2 cKO embryos and hyperactivation of pAkt does not rescue the trabeculation phenotype in Tie2 cKO embryos. The authors observe a decrease in Cyclin D1, cMYC, and pYAP in Tie2 cKO endocardial cells, suggesting that endocardial proliferation is inhibited. However, overexpressing cMyc in endocardial cells does not rescue the trabeculation defect in Tie2 cKO embryos. scRNA-seq identified an increase in Vcan expression in Tie2 cKO embyros, which is validated with a Vcan antibody. However, cleaved Vcan staining is decreased. The authors also observed upregulation of Adamts1 and Timp3 in the scRNA-seq data, and identify FoxO1 binding sites in Timp3 that they validate by ChIP-seq in cardiac endothelial cells. They propose a model whereby FoxO1 activity is regulated by Tie2 signalling through the PI3K/Akt pathway. FoxO1 then directly regulates Timp3 expression to regulate Vcan cleavage, thereby affecting cardiomyocyte proliferation and leading to the observed trabeculation defect.

The findings presented by the authors are novel and interesting, and the proposed mechanism significantly advances our understanding of the mechanism by which Tie2 signalling leads to trabeculation defects. However, there are some holes in the proposed mechanism that should be addressed to strengthen this claim.

© 2026. Published by The Company of Biologists under the terms of the Creative Commons Attribution License (https://creativecommons.org/licenses/by/4.0/).

Response: We appreciate the reviewer's comments recognizing the novelty and quality of the findings in this study.

SUGGESTIONS TO AUTHORS
Major:
1. Is each data point in the various quantification plots from an independent embryo? The quantification section in the methods mentions "six embryos of each genotype" were analysed, but all plots only have five data points. Please clarify.

Response: We thank the reviewer for noting this issue and have corrected as "five embryos of each genotype" in the methods section.

2. Line 187 refers to fig 4, but the data is in fig 3. Please correct. It is also not clear what control was used in this experiment, please clarify their genotype.

Response: We are sorry for the typo and thanks for the correction (Fig. 3F-H). The genotype of the control has been clarified in the figure legend.

Presumably the double cKO embryos examined in fig 3 (at E14.5) have a mild phenotype as the authors say that the majority die by E13.5 (line 182). Did the authors examine the phenotype in E13.5 double cKO embryos? Is the Tie2 phenotype also milder in those younger double cKOs? The E13.0 double cKO in fig S4B looks more severe than the E14.5 embryo shown in fig 3F. It would be good to include more data at the earlier stages to get a better sense of how much of the Tie2 phenotype one can attribute to FoxO1 activation and how much is due to other effectors of Tie2 signalling.

Response: Yes, 25% double knockout *Nfatc1Cre;Tie2*$^{f/f}$*;FoxO1*$^{f/f}$ animals have a mild phenotype (Fig. 3F-H). However, the majority of double knockout embryos never survived beyond E13.5. We had examined more than 10 of these double cKO embryos at E13.5 and found their trabeculation defects are very similar to *Tie2-cko* embryos (Qu, et al. 2019). So, we do not think it's necessary to include these data. We are sorry for the confusion, but the E13.0 mutant in fig S4B is actually a *FOXO1*$^{EC\text{-}CA}$ (which usually had more severe phenotype than *Tie2-cko*), instead of a double cKO.

3. I'm confused how the trabeculation defect observed in the PI3K hyperactivation embryos in fig 5 fits with the rest of the manuscript. Presumably the lethality/trabeculation defect in these embryos is not due to the proposed FoxO1/Timp3/Vcan pathway as one would expect FoxO1 to be phosphorylated and localised to the cytoplasm in these embryos (as in WT controls). Did the authors examine FOXO1 phosphorylation and/or localisation in the PI3K hyperactivation embryos?

Response: We thank the reviewer for this thoughtful comment. In this manuscript, we aim to present data to support the proposed FoxO1/Timp3/Vcan pathway mainly via inactivation of TIE2/reduced PI3K activation. Further, figure 5 shows that excessive PI3K signaling in EdCs also resulted in reduced cardiac trabeculation and embryonic lethality, suggesting that PI3K activation has to be finely modulated. We agree that "the lethality/trabeculation defect in these embryos is not due to the proposed FoxO1/Timp3/Vcan pathway as one would expect FoxO1 to be phosphorylated and localized to the cytoplasm in these embryos (as in WT controls)", which is supported by our recent examination of FOXO1 phosphorylation and/or localization in the PI3K hyperactivation embryos (added in new supplemental fig.9). Although PI3K hyperactivation did not lead to impaired versican cleavage, it resulted in hypotrabeculation via abnormal endocardial cell growth. It is very common that hypotrabeculated mutants exhibit severe heart failure and embryonic lethality because trabeculae have a 'prosurvival' role in cardiogenesis. Conversely, an excessive number of abnormal trabeculae do not often lead to early embryonic lethality but left ventricular non-compaction (like *Mib1*, NOTCH pathway regulator).

4. Line 473 in the discussion mentions the use of the ChIP-qPCR experiments to "validate that TIMP3 is a direct target of FOXO1 in developing endocardium". However, an important caveat here is that these experiments were performed using a cardiac endothelial cell line rather than endocardial cells. While I understand there are technical challenges to performing this experiment with endocardial cells, the fact that the validation of Timp3 and as a direct target of FoxO1 was not performed in endocardial cells should be made clear in the discussion.

© 2026. Published by The Company of Biologists under the terms of the Creative Commons Attribution License (https://creativecommons.org/licenses/by/4.0/).

Response: We thank the reviewer for this comment. To avoid over-interpretation, we have revised this sentence to "To further validate that TIMP3 is a direct target of FOXO1 in ECs, ---"

5. The model in fig 9 proposes that cardiomyocyte proliferation is downstream of the proposed Tie2/FoxO1/Timp3/Vcan pathway. However, cardiomyocyte proliferation was not examined in any of the experiments in this manuscript. I assume this point is a reference to the increased proliferation the authors have previously reported in Tie2 cKOs. Have the authors examined cardiomyocyte proliferation/cell number in the CA-FOXO1 or double cKO embryos? Direct evidence linking the proposed pathway to cardiomyocyte proliferation is lacking and this should be made clear in the model. The authors may already have the samples to perform such an analysis that would test this claim.

Response: We thank the reviewer for these thoughtful comments. Yes, we had examined proliferation of cardiomyocytes and EdCs in the CA-FOXO1 embryos, although only EdC proliferation data were shown in Fig. 3. Now cardiomyocyte proliferation data has been included as Fig.5E-G in new supplemental Fig.5. We did not checked proliferation of cardiomyocytes in double knockout *Nfatc1Cre;Tie2*$^{f/f}$*;FoxO1*$^{f/f}$ embryos, because the majority of double knockout embryos displayed very similar trabeculation defects as *Tie2-cko* embryos (Qu, et al. 2019).

Minor:
1. Please consider reporting P-values in full to allow for more accurate interpretation of results (see https://www.nature.com/articles/d41586-019-00857-9).

Response: All individual *p*-values have been included except when $P < 0.001$.

2. I understand the desire to present as much data as possible, and the authors have generated a lot of it, but nine figures is extensive and the main findings are well represented in figs 1, 3, and 7. Would the authors consider moving some of the other figures to the supplement? E.g., fig 2 and 4, and maybe even 5 and 6.

Response: Respectfully, we would like to keep these figures them in the main body of the paper as they do not exceed the figure limit for Development. We believe these figures are essential for interpretation of the results of the main paper and make our paper relatively easy to read. In addition, certain supplement materials might be only temperately available to the public readers online.

3. It is unclear to me what the significance is of the E16.5 control data in figure 1E. Given that the Tie2-cKO embryos do not survive past E13.5.

Response: Although the Tie2-cKO embryos do not survive past E13.5, the E16.5 control data in figure 1E showing more nuclear localization of FOXO1 at E16.5 than at earlier embryonic stages in WT, is consistent with the hypothesis that nuclear localization of FOXO1 inhibits EC proliferation.

4. It isn't clear what is meant by the sentence on line 136, "although no significant changes were detected on YAP expression". Please clarify this statement and what data this refers to.

Response: YAP immunostaining data has been included in supplemental material (new Fig. S4).

5. In table S3, should the FOXO1 CA/CA genotype be CA/+? Line 155 refers to mutants, I assume this means heterozygotes rather than homozygotes? Similarly, the Pik3ca H1047R and Nfatc1 Cre embryos in table S3 would all be heterozygotes, no? Please clarify.

Response: We thank the reviewer for noting this and we are sorry for the typos. In table S3, the genotype of control is FOXO1CA/+ instead of FOXO1CA/+. Line 155 refers to *Nfatc1*^{Cre};*FOXO1*$^{CA/+}$ mutants. Similarly, the Pik3ca H1047R/+ and Nfatc1 Cre; Pik3ca H1047R/+ embryos in table S3 all are heterozygotes. Clarifications have been made in the revised manuscript accordingly.

© 2026. Published by The Company of Biologists under the terms of the Creative Commons Attribution License (https://creativecommons.org/licenses/by/4.0/).

Second decision letter

MS ID#: dev.205674R1

MS TITLE: Endocardial Tie2 orchestrates cardiac trabeculation via sequestering FOXO1 within the cytoplasm and stimulating versican cleavage

AUTHORS: Xianghu Qu, H. Scott Baldwin, Kevin Tompkins, Yan Gong, Bryan Helm and Qi Liu

Dear Dr Qu,

I am happy to tell you that your manuscript has been accepted for publication in Development, pending our standard publication integrity checks.

© 2026. Published by The Company of Biologists under the terms of the Creative Commons Attribution License (https://creativecommons.org/licenses/by/4.0/).
